# Intercontinental trials reveal stable QTL for Northern corn leaf blight resistance in Europe and in Brazil

**DOI:** 10.1007/s00122-020-03682-1

**Published:** 2020-09-30

**Authors:** Ana L. Galiano-Carneiro, Bettina Kessel, Thomas Presterl, Thomas Miedaner

**Affiliations:** 1 State Plant Breeding Institute, University of Hohenheim, Stuttgart, Germany; 2 Kleinwanzlebener Saatzucht (KWS) KWS SAAT SE & Co. KGaA, Einbeck, Germany

## Abstract

**Key message:**

NCLB is the most devastating leaf disease in European maize, and the introduction of Brazilian resistance donors can efficiently increase the resistance levels of European maize germplasm.

**Abstract:**

Northern corn leaf blight (NCLB) is one of the most devastating leaf pathogens in maize (*Zea mays* L.). Maize cultivars need to be equipped with broad and stable NCLB resistance to cope with production intensification and climate change. Brazilian germplasm is a great source to increase low NCLB resistance levels in European materials, but little is known about their effect in European environments. To investigate the usefulness of Brazilian germplasm as NCLB resistance donors, we conducted multi-parent QTL mapping, evaluated the potential of marker-assisted selection as well as genome-wide selection of 742 F_1_-derived DH lines. The line per se performance was evaluated in one location in Brazil and six location-by-year combinations (= environments) in Europe, while testcrosses were assessed in two locations in Brazil and further 10 environments in Europe. Jointly, we identified 17 QTL for NCLB resistance explaining 3.57–30.98% of the genotypic variance each. Two of these QTL were detected in both Brazilian and European environments indicating the stability of these QTL in contrasting ecosystems. We observed moderate to high genomic prediction accuracies between 0.58 and 0.83 depending on population and continent. Collectively, our study illustrates the potential use of tropical resistance sources to increase NCLB resistance level in applied European maize breeding programs.

**Electronic supplementary material:**

The online version of this article (10.1007/s00122-020-03682-1) contains supplementary material, which is available to authorized users.

## Introduction

Maize (*Zea mays* L.) is the worldwide most productive crop with 1.12 harvested billion metric tons in 2018/19 (USDA/IPAD 2020). Projections estimate more than 183 million metric tons production growth in the next decade (OECD/FAO 2019) seeking to attend the increasing demand for food, feed and fuel. In Europe, especially in Germany, maize production increased exponentially since the end of the 1970s and maize is nowadays the second largest crop in acreage, where about 85% of the production is designated to silage and biogas maize and 15% to kernel maize (Bundessortenamt 2019). NCLB was firstly observed in southern Germany in 1995 (Welz et al. 1996; Welz 1998; Hanekamp 2016) and is nowadays the most devastating maize leaf disease in the country. On a worldwide basis, harvest losses by NCLB can vary from 15 to more than 60%, especially in tropical and subtropical environments (Raymundo and Hooker 1981; Tefferi et al. 1996; De Rossi et al. 2010; Cramptom 2015; Nwanosike et al. 2015; Romero 2016). Likewise, NCLB infections can lead to a reduction in silage digestibility and pre-disposition to stalk rot, representing a significant threat to farmers and seed growers (for review, please, refer to Galiano-Carneiro and Miedaner 2017). NCLB is caused by the ascomycete *Setosphaeria turcica* (Luttrell) Leonard & Suggs (anamorph: *Exserohilum turcicum* (Pass.) Leonard & Suggs. syn. *Helminthosporium turcicum* Pass., Boln Comiz.) which grows preferably in temperatures between 15 and 28 °C and in high humidity conditions. These conditions are primarily fulfilled in the subtropics, especially in the south of Brazil. This region represents one of the most important maize production regions in Brazil, the third largest maize producer country worldwide (CONAB 2020). However, it has also the ideal environmental conditions for NCLB infections including long periods of dew, long nights and average temperatures (INMET 2020) exactly in the optimal range of the disease development. These favorable conditions in addition to the presence of *S. turcica* can likely trigger NCLB epidemics. With frequent epidemics, the *S. turcica* populations grow concomitantly to the potential number of pathogen mutations leading to a strong selection pressure on both, pathogen and host. This strong NCLB pressure presumably occurred in many growing seasons in South Brazil leading to more complex *S. turcica* races (Navarro, personal communication) and highly resistant host plants due to maize breeders’ efforts (e.g., cultivar CDL 15, Kaefer et al. 2017).

In contrast, the three factors contributing to an epidemic (favorable environment, susceptible host and virulent pathogen) meet only sporadically in Europe. In addition, most probably due to the more recent history of the fungus in Europe, firstly recorded as an epidemic in the beginning of the 1990s in Austria (Hanekamp 2016), there was less interest for selection on highly resistant host plants, although genetic variation for NCLB resistance in Europe is present (Welz et al. 1999; Van Inghelandt et al. 2012). As fungicide application is expensive and laborious in the later stages of maize development, resistance breeding is the most economic and environmentally friendly way to reduce damage caused by *S. turcica*.

In the maize/*S. turcica* pathosystem, qualitative (race-specific) as well as quantitative, race-nonspecific resistances are known (Galiano-Carneiro and Miedaner 2017). In Europe, mainly race-specific resistances genes such as *Ht1*, *Ht2*, *Ht3* and *HtN (“Ht” refers to Helminthosporium turcicum*, former name of the pathogen*)* have been harnessed in applied breeding programs. This is expected as *Ht* genes are of practical use for breeders because the introgression of one *Ht* gene can potentially confer high resistance levels; however, this resistance can be quickly overcome by virulent pathotypes. Historically, *Ht1* described in the 1960s was the longest effective resistance gene compared to the other *Ht* genes. However, in the 1970s, race 1 overcame *Ht1* making the resistance ineffective in areas where race 1 is abundantly present (Bergquist and Masias 1974; Welz 1998). Gene pyramiding, *i.e.,* stacking multiple *Ht* genes in one genotype, is a well-known approach to increase the durability of resistance genes (Sánchez-Martín and Keller 2019). However, the emergence of complex races such as the race 123 N firstly identified in the Heilongjiang region in China (Ma et al. 2020) in addition to the strong directional selection for pathogen virulence when large acreages are sown may render also gene pyramiding ineffective (Pilet-Nayel et al. 2017).

In Brazil and in Europe, the distribution of these races is usually region specific. To exemplify this, the predominant race in Castro was race 1, while races 0 and 2 were more frequently observed in Ponta Grossa in a race monitoring conducted in 2019 (Navarro, personal communication). In Europe, races 3 N and 3 were the most common in southwest of France and north of Italy, while race 1 was the most abundant in Austria, Hungary, and the German Upper Rhine region between 2011 and 2012 (Hanekamp 2016). These race monitorings illustrate that most of the monogenic resistances mediated by *Ht* genes have already been overcome by virulent pathotypes. Therefore, race monitoring is an important tool to assist breeder’s decision on the choice of the *Ht* gene to apply in each region. Moreover, these examples demonstrate that breeding for quantitative, race-nonspecific resistances should be prioritized in the NCLB pathosystem.

Genetic resources can be exploited to identify new sources of resistance alleles that can potentially increase durability of host resistance (McDonald and Linde 2002; Mayer et al. 2017). Highly resistant Brazilian maize lines with quantitative resistances to NCLB have already been identified in Brazil (Kaefer et al. 2017; Ribeiro et al. 2016) and introducing NCLB resistance from Brazilian genotypes to Europe can be a great opportunity to increase NCLB resistance levels, but little is known about the effect of these resistance sources in European environments. To investigate the potential use of Brazilian sources, three resistant Brazilian donors were each crossed with adapted elite double haploid (DH) European lines. These donors are elite lines from KWS SAAT SE & Co. KgaA breeding programs and employed here for the first time in a NCLB study. As our objective was to exploit quantitative resistance to NCLB, none of the parental lines neither the testers possessed the *Ht* genes *Ht1*, *Ht2* and *Htn1* according to markers developed and analyzed by KWS SAAT SE & Co. KGaA. To potentially discover NCLB resistance, which is durable and stable across many environments, we performed QTL mapping with a total of 742 DH lines and their respective testcrosses assessed for NCLB resistance across two locations in Brazil and 11 location-year combinations (= environments) in Europe (Austria, France, Germany and northern Italy), considering both line per se and testcross assessment. This project was a part of an applied maize breeding program, and analyzing the maximum number of genotypes and environments was desired.

In particular, our objectives were to: (1) test the potential use of Brazilian resistant germplasm to tackle NCLB infection in European conditions; (2) assess quantitative-genetic parameters for NCLB resistance in per se and testcross doubled haploid (DH) populations; (3) analyze the genetic architecture of NCLB resistance by multi-parent QTL mapping and biparental QTL mapping; (4) assess genomics-assisted breeding strategies for an efficient introgression of NCLB resistance in adapted plant materials.

## Materials and methods

### Plant material and field trials

This study comprised biparental populations derived from three tropical donors (T1, T2, T5, abbreviated T) from Brazil selected to be highly resistant to NCLB. They were crossed with seven susceptible elite lines adapted to Europe (A) resulting in the following seven biparental populations: T1 × A1, T1 × A2, T1 × A10, T2 × A3, T2 × A4, T2 × A5 and T5 × A11. (Suppl. Figure 1). Four crosses derived from resistant donors T1 and T5 belong to the stiff-stalk synthetic (SSS) heterotic group and three crosses derived from resistant donor T2 to the non-stiff-stalk (NSS) heterotic group. Crosses resulted in 22–148 DH lines per population summing up to 742 unique F1-derived DH lines. Subsequently, the DH lines were crossed with line testers moderately to highly susceptible for NCLB. The testers belonged to the respective opposite heterotic group in Brazil. In Europe, one susceptible flint tester was crossed with DH lines belonging to both SSS and NSS heterotic groups to shorten maturity for the cooler European conditions. All genotypes are proprietary materials of KWS SAAT SE & Co. KgaA. Segregating plant material is available on request to this company for scientists without any commercial interest. A respective MTA must be signed in advance.

Populations showing common parents were randomized together to increase the accuracy of entry comparison (Piepho et al. 2006a). This led to four trials: (1) “trial 1″, composed by all individuals from populations T1 × A1, T1 × A2 and T1 × A10; (2) “trial 2,” composed by all individuals from populations T2 × A3 and T2 × A4; (3) “trial 3,” composed by population T5 × A11; (4) “trial 4,” composed by all individuals from population T2 × A5, randomized separately from “trial 2” for seed logistic reasons. Populations composing trials 1 and 3 belonged to the SSS, while populations from trials 2 and 4 belonged to the NSS heterotic group.

Populations were evaluated for NCLB in the growing seasons 2019 in Brazil and 2017, 2018 and 2019 in Europe (Suppl. Figure 1). Per se performance was evaluated in one location in Brazil and four locations in up to three years (in total six environments) in Europe. Testcrosses were evaluated in two locations in Brazil and in 7 locations and up to 2 years (in total ten environments) in Europe (Supplementary Table 1). The testing environments in Brazil and in Europe will be referred as different continents for simplification. Trials were allocated in alpha-lattice designs with two replications per location, except for per se evaluation in Brazil for trials 1 and 2, and testcross evaluation for trial 3 in Europe; trials were allocated following a p-rep design where about 80% of the data was replicated to efficiently allocate the limited number of harvested seeds. Resistant and susceptible checks comprising KWS SAAT SE & Co. KgaA property DH lines and hybrids in addition to parental lines were sown in each location leading to at least eight common genotypes among trials. Testcrosses from trials 1 and 2 were evaluated in both the South of Brazil and Europe (Austria, France, Germany and Italy). Trial 2 was tested for line per se performance in both continents.

In addition to NCLB, we assessed female flowering (FF) time and plant height (PH) in Europe. These traits were assessed in alpha-lattice designs with one replication in Monselice, Italy, in 2018 and 2019, and with two replications in Neupotz, Germany, in 2018, resulting in three European environments (Supplementary Table 1).

Our experimental unit was a two-row observation plot with a length of 4.0 m and a distance between rows of 0.5 m in Brazil and one row observation plot with the same dimensions in Europe. In Europe, disease spreader rows for artificial inoculation were additionally planted in the fields between each second, fifth, seventh or tenth row depending on the field location. All entries were treated according to local best agronomic practices not affecting the development of NCLB.

### *Setosphaeria turcica* inoculation and trait assessment

All environments in Europe and Ponta Grossa (PG) in Brazil were inoculated with *S. turcica* warranting uniform inoculum distribution. Leaves with NCLB symptoms from the respective location were collected the year before each testing season, air-dried and stored until inoculation. A parallel project was conducted to identify the races present in each field location. Differential lines were employed for the race identification, and in most of the locations, several *S. turcica* races were present (Navarro, personal communication). Subsequently, symptomatic leaves were crashed, and 1 g of the inoculum was added to the maize whorl of the spreader rows in Europe. In Brazil, the first and the last two plants of each row were inoculated. This procedure was conducted at the vegetative stage of 12–14 true visible leaves (V12–V14) and latest 10 days before tasseling as originally proposed by Hooker (1973).

**Fig. 1:**
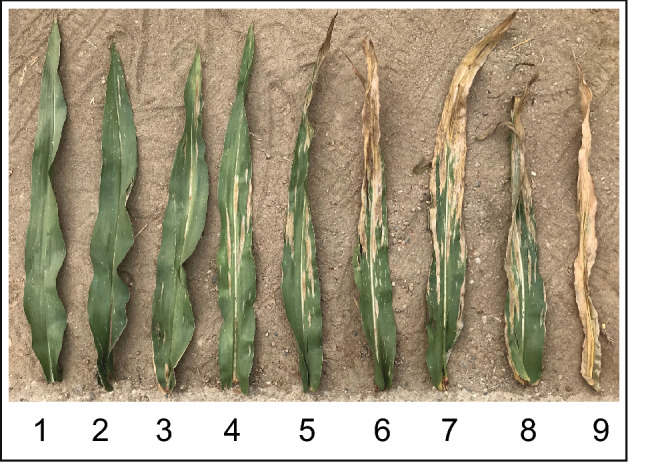
NCLB damage scale 1–9, where one is a plot without NCLB symptoms and nine is a plot fully diseased, represented by one single leaf in this figure

About 120 days after sowing, at the phenological stage R5 to R6, the NCLB symptoms were visually assessed in a plot-wise severity scoring scale ranging from 1 to 9, where 1 = entire plot without NCLB symptoms and 9 = entire plot fully diseased (Hurni et al. 2015; Fig. 1). The NCLB plot-wise rating was assessed two to four times in an interval of 27–91 days post-inoculation where the first inoculations took place in the beginning of January in Brazil and end of June in Europe (dpi; Supplementary Table 2). The average (NCLB_m_) and the final/last NCLB visual scoring (NCLB_f_) of all evaluations were considered for further analyses.

Plant height was assessed by measuring the size of a representative plant per experimental unit from the soil level to the beginning of the tassel bifurcation. Female flowering was measured as the number of days from sowing to the day that at least 50% of the row had extruded silks.

### Phenotypic data analysis

Phenotypic analyses for single environments were performed using linear mixed models and outlier detection procedures as proposed by Bernal-Vasquez et al. (2016). Combined analysis without a maximum of 15% outliers, comprising rows with lodging plants or rows with low plant density were conducted according to the mixed model:

$$ y_{ijyklm} = \mu + G_{i} + Y_{j} + T_{y } + L_{k} + LY_{kj} + LT_{ky} + LTY_{kyj} + LTYR_{kyjl} + LTYRB_{kyjlm} + e_{ijyklm} . $$

where $\mu$ represents the overall mean, $G_{i}$ the effect of the $i$ th genotype, $Y_{j}$ the effect of the *j*th year, $T_{y }$ the *y*th trial, $L_{k}$ the effect of the *k*th location, $m^{ith}$ B_m_ the incomplete block, and its interaction terms ($LY_{kj} ,LT_{ky} ,  LTY_{kyj,}  LTYR_{kyjl} and LTYRB_{kyjlm}$), and $e_{ijyklm}$ the heterogeneous error variance. The same model excluding the location and year effects was employed for the single location analysis.

$G_{i} ,  Y_{j}$ and $T_{y }$ effects were included in the fixed statement of the model to obtain the best linear unbiased estimators (BLUEs). The variance components were obtained through the restricted maximum likelihood method (REML) by including only the $Y_{j}$ and $T_{y }$ effects in the fixed statement of the model above. All other effects were included in the random statement of the model. The significance of the variance components was obtained by likelihood ratio test between the full and incomplete model (Stram and Lee 1994). Binary dummy variables were used to separate the effects of each population, checks and replicates as proposed by Piepho et al. (2006b). For the sake of simplicity, dummy variables were not shown in the model above.

The broad-sense heritability (*H*^2^) was estimated following Cullis et al. (2006):

$$ H^{2} = 1 - \frac{{\bar{\vartheta }_{\text{BLUP}} }}{{2\sigma_{G}^{2} }} $$

where $\bar{\vartheta }_{\text{BLUP}}$ is the mean variance of a difference of two BLUPs and $\sigma_{G}^{2}$ is the genotypic variance.

Corrections for flowering date were conducted according to the approach of Emrich et al. (2008). The female flowering scorings were added in the fixed statement of the mixed models to obtain the NCLB_f__FF.

Phenotypic correlations based on BLUEs of female flowering time, plant height and NCLB severity as well as the correlation of the NCLB severity between trials in the two continents were calculated with Pearson product moment correlation coefficients.

The relative efficiency of indirect selection using line per se to predict testcross performance was obtained by the following equation proposed by Falconer and Mackay (1996) and reviewed by Löffler et al. (2011):

$$ {\text{RE}} = \frac{{H_{PS} \times r_{G} }}{{H_{TC} }} $$

where RE is the relative efficiency, $H_{PS}$ is the square root of the per se heritability, $H_{TC}$ is the square root of the testcross heritability, and $r_{G}$ is the genetic correlation between line per se and testcrosses.

The genetic correlation ($r_{G}$) between per se and testcross was obtained using the following formula proposed by Cooper et al. (1994):

$$ r_{G} = \frac{{r_{P} }}{{\sqrt {H_{TC} \times H_{PS} } }} $$

where $r_{P}$ is the phenotypic correlation between per se and testcross, $H_{TC}$ is the square root of heritability of testcrosses, and $H_{PS}$ is the square root of heritability of lines per se.

All analyses were conducted within the R environment (R Development Core Team 2018, version 3.5.1). Mixed-model computations were performed using the R package ASReml-R 3.0 (Gilmour et al. 2009).

### Molecular data

All DH lines were genotyped at KWS molecular laboratory using an Illumina 15 k SNP chip based on the public Illumina MaizeSNP50 BeadChip. All ten chromosomes were partitioned into bins of 0.5 cM according to the public genetic map IBM and the physical map AGPv02 (Ganal et al. 2011); therefore, we call the positions “putative cM” (putcM). Regions adjacent to centromeres were especially markedly enriched to account for the low recombination rates in this area.

The number of polymorphic markers in each population ranged from 5 to 6 k. Quality control was conducted by removing monomorphic or missing alleles for both parents, heterozygous genotypes at the parents, genotypes with more than 25% missing values, markers with more than 10% missing data and markers with minor allele frequency (MAF) lower than 5% in each population. After the quality check, 1454, 3223 and 2212 SNP markers were available for donors T1, T2 and T5, respectively.

### Multi-parent QTL mapping analysis (bi-allelic model)

The T1 and T2 donor groups comprised populations that were connected through the respective resistant tropical parent. They were allocated as following: “Donor T1,” comprised the individuals from populations T1 × A1, T1 × A2 and T1 × A10, included in the trial 1; “Donor T2,” comprised the individuals from populations T2 × A3 and T2 × A4, included in the trial 2, and “Donor T5” comprised population T5 × A11, included in the trial 3. The population T2 × A5 did not show significant genetic variance for both NCLB traits; hence, it was not integrated into the analyses.

Multi-parent QTL mapping analysis was conducted with the R package mppR version 1.2.0 (Garin et al. 2018). Succinctly, multiple biparental populations that were connected through one resistant tropical parental, as donors T1 and T2, were analyzed jointly by the method of composite interval mapping (CIM) (Zeng 1993, 1994).

The additive effect of the QTL was obtained through the bi-allelic model of the package mppR. This model considered that alleles from different populations with the same SNP were identical by state (IBS) (e.g., model B in Würschum et al. 2012; Garin et al. 2017). To avoid false positives, population structure (Supplementary Fig. 2) was accounted by the k-model proposed by Yu et al. (2006).

QTL significance thresholds were obtained by permutation tests performing 1000 iterations (Broman and Sen 2009). QTL mapping for each model was conducted in a first step by a simple interval mapping (SIM) and the significant QTL from this analysis were applied as cofactors for the CIM. The confidence interval of each QTL was obtained by –log10 (*p*) value drop off interval. The contribution of each QTL to the phenotypic variance was computed by comparing the full, containing all the QTL, and incomplete models, excluding only the detected QTL of interest. Individual explained genotypic variance ($$ p_{G} ) $$ was obtained following the equation proposed by Utz et al. (2000):

$$ p_{G} = \frac{{R_{\text{adj}}^{2} }}{{H^{2} }} $$

where $R_{\text{adj}}^{2}$ corresponds to the adjusted $R_{{}}^{2}$ that was adjusted for the number of parameters included in the linear model and $H^{2}$ the average broad-sense heritability of each population composing a donor group.

### Biparental QTL mapping

Donor T5 was calculated with the CIM QTL mapping function implemented in the R package RQTL because only one population was available for this donor (T5 × A11) (Broman et al. 2003). The QTL significance threshold was defined by 1000 iterations permutation test (Broman and Sen 2009). Five markers were forward selected and used as covariates in the Haley–Knott regression (Haley and Knott 1992). Additive effects per parent component, global and partial explained phenotypic variance and QTL confidence interval were computed as described in the previous section. Each identified QTL was ordered by the type of material assessment (line per se or testcrosses) and received the nomenclature “*qx*” where “*x*” is a consecutive number of QTL. Same nomenclature indicates that QTL are co-located. However, QTL peaks identified within a large confidence interval are more likely to have many co-located QTL. In addition, the chromosome location of each identified QTL was described in chromosome bins. This refers to the interval that contains all loci delimited by two core markers. For example, QTL q4 is present on chromosome 7, region 7.03 of the maize genome within 128,175,453–156,050,469 bp (for more details, please, refer to MaizeGDB).

### Marker-assisted, genomic and weighted genomic predictions

Marker-assisted predictions (MAS) were conducted with the significant QTL explaining more than 5% of the genotypic genetic variance for the trait NCLB_f_ within donors T1, T2 and T5. Genomic prediction was carried out by ridge-regression BLUP (RR-BLUP, Whittaker et al. 2000) with the R package “rrBLUP” (Endelman 2011; Endelman and Jannink 2012) within each donor group. Missing SNP marker information was imputed for each donor group with the software LinkImpute (Money et al. 2015) and resulted in high imputation accuracies (> 97%). In addition, we performed a weighted ridge-regression BLUP (wRR-BLUP) where QTL explaining more than 5% of the genotypic variance was added to the fixed statement of the genomic prediction model (Bernardo 2014; Zhao et al. 2014; Spindel et al. 2016). The main objective of this approach is to increase the frequency of the major effects within the breeding population (Bernardo 2014). In addition, this model has been proofed to increase the prediction accuracies in different crops (Gaikpa et al. 2020; Galiano-Carneiro et al. 2019; Herter et al. 2019).

The performance of the MAS, RR-BLUP and wRR-BLUP were evaluated by a five-fold cross-validation (CV) procedure. The data were randomly divided in five different folds where 80% of the data comprising phenotypic and molecular data were employed in the training set to predict the phenotypic values of the remaining 20% data, comprising only the molecular data, in the prediction set to assess the prediction error (Utz et al. 2000). This procedure was repeated 200 times (i.e., 1000 cross-validations), each repetition with a random composition of folds to assess CV error. For each fold composition, prediction ability was calculated as the Pearson’s correlation between predicted versus observed values for each evaluated model. This procedure was also employed to compare the prediction ability of different family compositions in the training and prediction sets. For this, 60 genotypes were composing the training set and the remaining genotypes comprised the prediction set. Prediction accuracy was the prediction ability divided by the square root of the trait broad-sense heritability, composed by the average H^2^ of families with common parent.

## Results

**Fig. 2:**
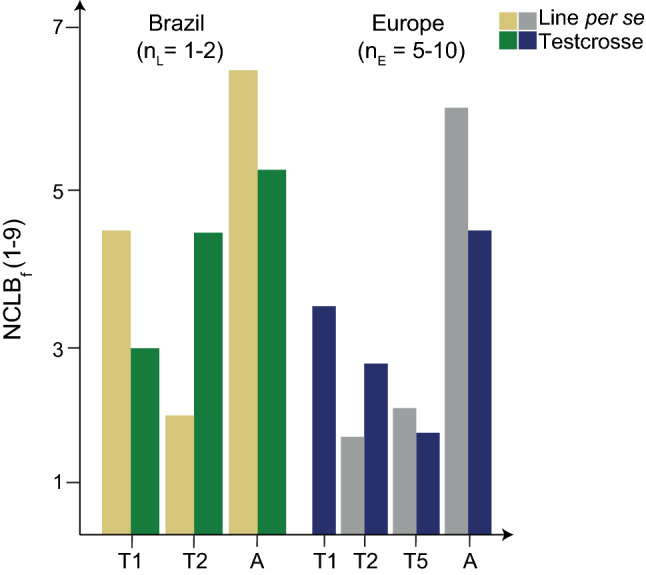
Entry means of each tropical donor (T1, T2 and T5) in comparison with the mean of the adapted parent lines (A) evaluated in line per se and testcross combinations for the NCLB_f_ in several locations (*n*_L_) in Brazil and environments (*n*_E_) in Europe

**Fig. 3:**
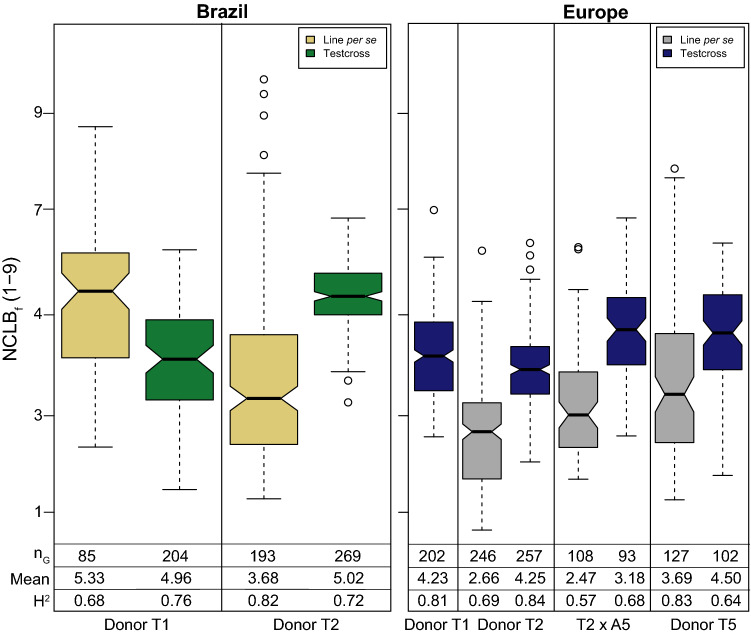
Notched boxplots for NCLB final score (NCLB_f_) evaluated as line per se and testcross in Brazil (**a**) and Europe (**b**) in a damage scale of 1–9

The tropical donors T1, T2 and T5 were considerably more resistant than the mean of the adapted elite lines for line per se and testcross performance (Fig. 2). In Brazil, disease severity was, on average, higher for both, lines per se and testcrosses, compared to Europe. However, we had a maximum of two locations in Brazil and only testcrosses were assessed for donor T1 (Fig. 3). In Brazil, the testcrosses of donor T1 were, on average, more resistant than the lines, while in contrast the testcrosses of donor T2 were more susceptible than the lines. In Europe, all testcrosses showed a considerably higher susceptibility than the respective lines. All populations showed moderate to high broad-sense heritabilities for both NCLB ratings ranging from 0.52 to 0.90. Adjusted means indicated a quantitative distribution of NCLB_f_ with mean severity scores ranging from 2.60 to 5.68 and high, significant (*P* < 0.001) genetic variance for NCLB_m_, NCLB_f_, FF and PH (Supplementary Table 3). For the sake of simplicity, we will refer in the following to the populations according to their tropical resistance donor, i.e., to populations T1 × A1, T1 × A2 and T1 × A10 as donor T1, to populations T2 × A3 and T2 × A4 as donor T2 and to population T5 × A11 as donor T5.

Correlations among traits were also positive and significant for both, lines per se and testcrosses (Supplementary Table 4), where the traits NCLB_f_ and NCLB_m_ had the highest positive correlation (*r* ≥ 0.90, *P* < 0.001) in both continents. Hence, we focus on NCLB_f_ to avoid redundancy. The line per se correlations between NCLB_f_ and FF were significant and moderate from *r* = − 0.40 to *r* = − 0.41 (*P* < 0.001) depending on the donor, while none of the correlations were significant between NCLB_f_ and PH. For the testcrosses, we observed in Europe a significant, but moderate negative correlation between NCLB_f_ and FF ranging from *r* = − 0.38 to *r* = − 0.52 (*P* < 0.001) depending on the resistance donor. The correlations between NCLB_f_ and PH were significant for all donors except for donor T5. The significant correlations ranged from *r* = − 0.13 (*P* < 0.05) to *r* = − 0.34 (*P* < 0.001) (Supplementary Table 4). Corrections for flowering date did not reduce the correlations between NCLB_f_ and FF.

**Fig. 4:**
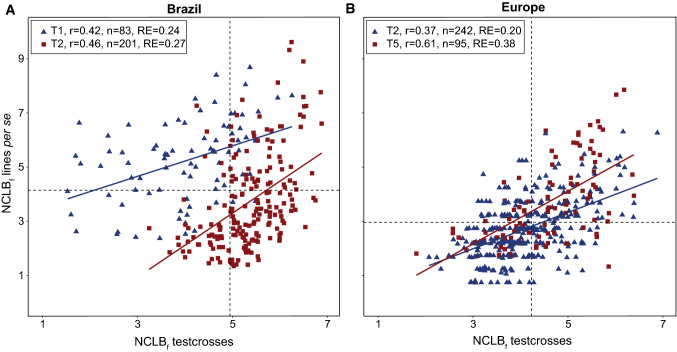
Scatter plots for final NCLB score (NCLB_f_) evaluated as line per se and testcrosses in Brazil (**a**) and in Europe (**b**) as well as the phenotypic correlation (*r*), number of genotypes (*n*) and relative efficiency (RE) of per se indirect selection for testcross performance. The dashed lines represent the mean of families for lines per se and testcrosses (4.68 and 4.98 in Brazil; 3.01 and 4.28 in Europe)

**Fig. 5:**
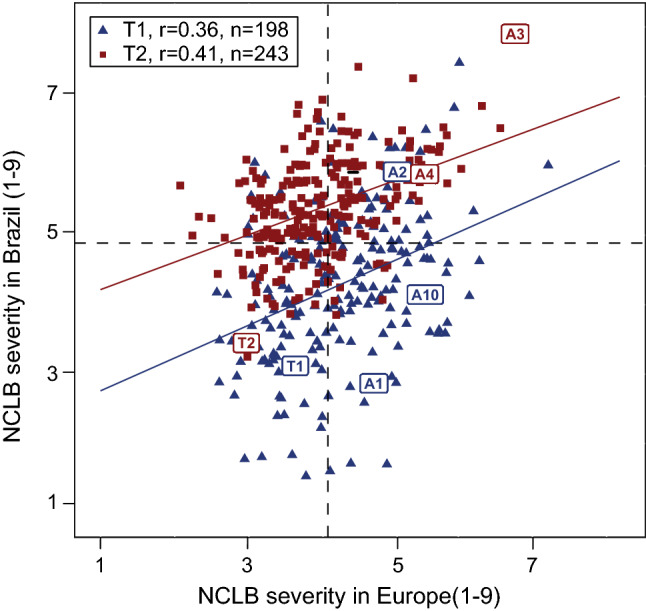
Scatter plot between Brazil and Europe for NCLB_f_ assessed for tropical donors T1 and T2. The dashed lines represent the mean of families within donors T1 and T2 scoring for each continent (4.98 for Brazil and 4.24 for Europe). Parent testcrosses were not included in the regression but are indicated in the labels

Lines and testcrosses showed moderate and positive correlations (*P* < 0.001) in Brazil and Europe (Fig. 4). Relative efficiencies of selecting testcross performance by per se performance were low in Brazil and Europe throughout. The efficiency was slightly higher for donor T5 compared to donor T2 in Europe (Fig. 4). Between Brazil and Europe, moderate phenotypic correlations of NCLB resistance for testcrosses were observed (*r* = 0.36 for donor T1 (*P* < 0.001), *r* = 0.41 for donor T2 (*P* < 0.001), Fig. 5).

**Fig. 6:**
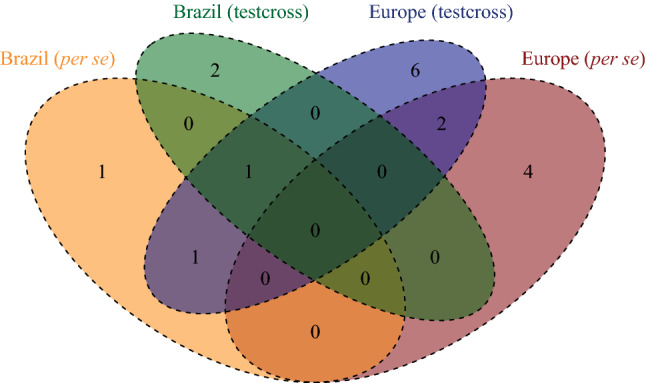
Venn diagram of the co-located QTL for NCLB_f_ score identified between and within continents

QTL mapping for NCLB_f_ resulted in one to four QTL each for per se and testcrosses depending on resistance donor and continent, where no QTL was identified for lines per se belonging to donor T1 (Table 1). The explained genetic variances per QTL ranged from 5.4 to 13.3% in Brazil and from 3.6 to 31.0% in Europe, while the tropical parents reduced NCLB_f_ damage from − 0.29 to − 2.48 scores considering both continents (Table 1; Supplementary Table 5). Four QTLs (q4, q5, q7 and q8) were co-located among per se and testcrosses within and between continents (Fig. 6).

**Table 1:** Number of QTL (*n*_QTL_) identified for the NCLB final score for each donor including the number of genotypes (*n*_G_) and markers (*n*_M_), minimum and maximum range of confidence interval (CI, putcM), explained genotypic variance range ($p_{G}$) and allele substitution effect (α-effects) for different models. For details of each identified QTL, please refer to Supplementary Table 5

Donor	*n*_G_	*n*_M_	Brazil				Europe				QTL model
			n_QTL_	CI (putcM)	$p_{G}$	α-effect	n_QTL_	CI (putcM)	$p_{G}$	α-effect	
*Per se*											
T2	236	3223	3	1.2.43/20.13	10.87/13.29	− 1.24/− 1.11	4	1.2/39.85	10.01/15.84	− 0.70/0.55	Bi-allelic
T5	129	2212	–	–	–	–	2	3.55/10.13	21.28/28.52	− 0.70/− 0.62	Biparental
*Testcrosses*											
T1	178	1454	2	1.91/111.14	8.45/12.41	− 0.65/0.52	3	3.86/209.86	3.57/18.57	− 0.57/0.54	Bi-allelic
T2	236	3223	1	10.81	5.43	− 0.30	4	8.19/22.85	7.10/24.02	− 0.74/− 0.34	Bi-allelic
T5	129	2212	–	–	–	–	3	8.19/18.24	15.75/30.98	− 0.42/− 0.29	Biparental

**Fig. 7:**
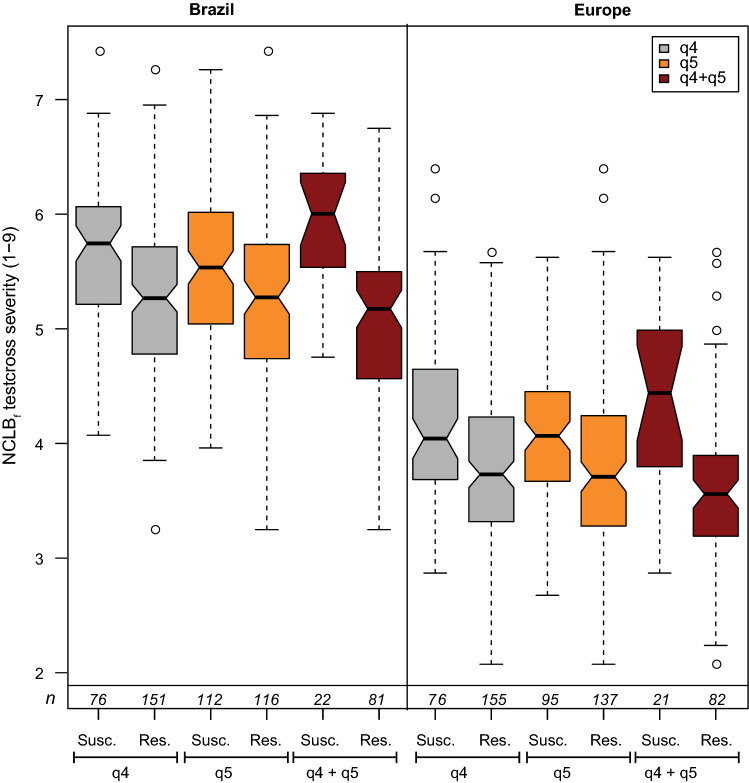
Notched boxplots of allelic effects for both environmentally stable NCLB QTL q4 and q5 and their combination for final NCLB score (NCLB_f_) severity (1–9) for donor T2 testcrosses

In Europe and Brazil, QTL q4 and q5 on chromosome bins 7.03 and 9.04 were identified within the same confidence interval range. QTL q4 explained 13.29% and 16.83% of the genotypic variance in Brazil and in Europe, respectively. QTL q5 explained 10.95% and 7.10% of the phenotypic variance in Brazil and in Europe, respectively, showing a significant reduction of NCLB severity, especially when both QTL are present (Fig. 7). The QTL q4 was identified on chromosome 7 at the physical position 153.88 Mbp and in the 155.11 Mbp in Brazil and Europe, respectively (Supplementary Table 5). QTL q5 was identified on chromosome 9 at the physical position 100.37 Mbp in Europe and at the positions 107.36 and 108.35 Mbp in Brazil as two QTLs were identified within the same confidence interval (Supplementary Table 5).

For the traits FF, we detected nine and for PH we detected five QTL on chromosomes 2, 7, 8, 9 and 10 (Supplementary Table 5). Among the QTL identified for FF, five were co-localized with NCLB_f_: q5, q6, q7, q14 and q16 which is in accordance to the moderate correlation between NCLB_f_ and FF ranging from − 0.38 to − 0.53 depending on resistance donor (Supplementary Fig. 3, Supplementary Table 5). Among the overlapping QTL, QTL q23 was coding the gene GRMZM2G067921 which is known to delay flowering time (Maize 2020). The tropical lines were flowering 16 days later and were 13–32 cm higher than the adapted lines, both measured in testcross combinations in Europe, according to the allele substitution effect of the identified QTL (Supplementary Table 5). One QTL, q7, was overlapping between PH and NCLB_f_.

**Fig. 8:**
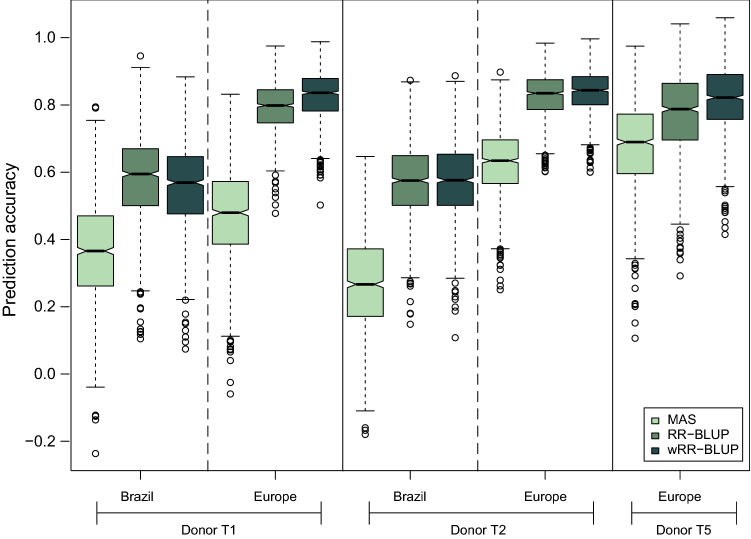
Prediction accuracies obtained from marker-assisted selection (MAS), genomic selection (RR-BLUP) and weighted genomic selection (wRR-BLUP) for each donor group and continent for testcrosses

Both genomic prediction methods (RR-BLUP and wRR-BLUP) showed higher prediction accuracies compared with marker-assisted selection (MAS, Fig. 8). Likewise, wRR-BLUP showed slightly higher prediction accuracies compared with standard RR-BLUP. Genomic prediction accuracies were estimated lower for Brazilian than for European environments (Fig. 8).

**Fig. 9:**
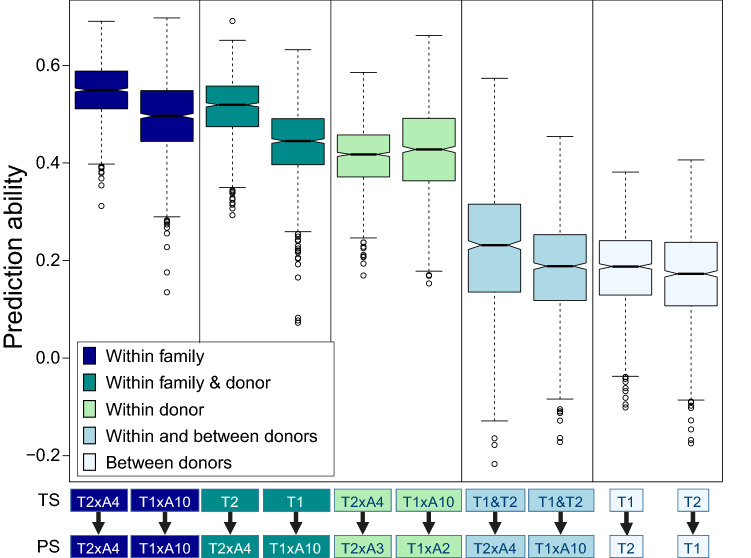
Prediction accuracies within and between families from the same and different heterotic groups (T1 belongs to stiff-stalk synthetic, T2 to non-stiff-stalk heterotic group)

The prediction ability was the highest when the training and the prediction sets comprised the same family. On the other hand, the prediction ability was the lowest when families from different heterotic groups were composing the training and the prediction sets (Fig. 9).

## Discussion

NCLB is one of the world’s most devastating leaf diseases in maize. Brazilian maize is a promising source of resistant genotypes, but little is known about the effect of these tropical resistance sources in European environments. Therefore, we investigated the potential use of Brazilian donors for NCLB resistance in the phenotype and molecular levels by conducting multi-environmental trials, QTL mapping and genomic prediction.

### Assessing Brazilian resistance donors in intercontinental trials

Tropical donors were tested as per se and testcrosses in Brazil and for the first time also in Europe. Our trials were conducted during the growing season of 2019 in two locations in the South of Brazil, where the environmental conditions are favorable for NCLB infections, and during the growing seasons of 2017, 2018 and 2019 in Europe on one to 10 environments (Supplementary Table 1). The locations represented the target areas of different grain maize maturity groups with the German locations being the earliest, followed by French and Italian locations as the latest ripening group (Rüdelsheim and Smets 2011; Czarnak-Kłos and Rodríguez-Cerezo 2010). Because of the highly differing maturity growing zones and different photoperiod response between Brazil and Europe, Brazilian donors could be tested only as testcross progeny in German and French locations.

The tropical donors, assessed both per se and in testcrosses, showed a moderate to high resistance level to NCLB in both continents, demonstrating that the Brazilian resistance sources are also resistant in the different maize maturity growing zones of Europe. However, lines per se originating from crosses between Brazilian resistance donors and susceptible European elite lines possessed higher average NCLB severity in Brazil than in Europe. This could be explained by more favorable environmental conditions for NCLB incidence in Brazil compared to Europe and/or more aggressive fungal populations. In Brazil, the average temperature between inoculation and the last field evaluation was 20.4 °C varying from 17.5 to 27.8 °C (INMET 2020, Supplementary Table 2). Considering all European locations, the average temperature between inoculation and the last field evaluation was 22.7 °C varying from 3.7 to 38.4 °C (Agrarmeteorologie Baden-Württemberg 2020; AgrarMeteorologie Bayer 2020; Ilmeteo 2020; Meteociel 2020; Time and date 2020), which indicates that the minimum and maximum temperatures in Europe were not within the ideal range of NCLB development of 15–25 °C (Carson 1999; Hanekamp 2016; Galiano-Carneiro and Miedaner 2017).

Race monitoring studies were conducted in the same field locations where our trials were located. In 2019, the predominant race in Castro was race 1 (*n* = 7 samples) and in Ponta Grossa races 0, 1, 12, 23 N and 2 occurred where races 0 and 2 were more frequently observed (*n* = 14) (Navarro, personal communication). Hanekamp (2016) collected leaf samples with NCLB symptoms from regions where our European trials were located in 2011 and 2012 and concluded that race 0 was the most abundant in the south of Germany and Austria, while race 3 N and 3 were the most common in southwest of France and north of Italy, while race 1 was the most abundant in Austria and Hungary and the German Upper Rhine region. These results indicate that a different *Setosphaeria turcica* race composition was observed in each of our field locations. Because we report in this study only QTLs identified across locations despite the different race compositions, we can conclude that these QTLs should be quantitatively inherited and not based on race-specific resistances.

The introgression of exotic, quantitatively inherited resistance QTLs by marker-assisted backcrossing (MABC) can be hampered by the lack of adaptation traits in Brazilian materials. Therefore, flowering time, which can restrict the employment of QTL in a broad number of environments, and plant height, which can lead to stalk lodging, were also assessed. These adaptation traits were solely assessed in one location in Germany in 2018 and in one location in Italy during the growing seasons 2018 and 2019. The correlations between the German and the Italian location for each donor group were high (*r* > 0.91, *P* < 0.0001; data not shown), demonstrating that the ranking of flowering time did not significantly change in contrasting environments and it was possible to compare flowering time with our NCLB ratings assessed in different environments.

The adapted elite lines assessed in testcross combination were maturing on average 16 days earlier than the tropical lines assessed in testcross combination in European environments according to the BLUEs of the parental lines (data not shown) and were, concomitantly, more susceptible than the donor lines. This justifies the negative correlations observed between NCLB_f_ and FF. This tendency resulted in a long flowering period of testcrosses (e.g., 34 days in Italy 2019), and this can partially explain why correcting for early flowering as described by Emrich et al. (2008) did not reduce the correlation between NCLB and FF (*r* = − 0.51 vs. *r* = − 0.67). Van Inghelandt et al. (2012) corrected the commercial maize germplasm employed in their study by dividing the genotypes in different maturity groups where the inoculation of late maturity group started 2 weeks later than the earliest group (Bormann et al. 2004). They conducted QTL mapping for the adjusted and non-adjusted NCLB scoring for flowering time and identified different QTL for each trait. Previous research projects investigating genetic architecture of NCLB resistance within adapted US and European materials revealed very low negative correlations between NCLB symptoms and FF ranging from − 0.06 to − 0.14 (Balint-Kurti et al. 2010) and a moderate negative correlation of 0.53 (Van Inghelandt et al. 2012). These results are in line with the correlations observed in our study. In addition, as *S. turcica* is a hemibiotrophic pathogen the disease is expected to advance faster in necrotrophic tissues (Van Inghelandt et al. 2012, Jiang et al. 1999).

Correlations between NCLB_f_ and PH also yielded negative values, indicating that short plants were more affected by NCLB. This is also mostly specific to our plant material since adapted lines assessed in testcross combination were on average 30 cm shorter than the tropical donors lines according to the BLUEs of the parental lines (data not shown). Moderate positive correlations between FF and PH were also observed, demonstrating that late and tall genotypes were less affected by NCLB, most probably because of the photoperiod sensitivity of the tropical donors.

### Implications for NCLB resistance breeding programs

Although genotype by year and genotype by location interactions played an important role in per se and testcross assessments, moderate to high heritabilities were observed both in Brazil and in Europe. This suggests that we were consistent with our visual scoring methodology and that our material had adequate genetic variation that can be exploited in maize resistance breeding programs of both continents.

The relative efficiency of indirect selection of lines per se for testcross performance ranged from 0.20 to 0.38 depending on the population and assuming the same selection intensity. The relative efficiency represents the expected correlated response of hybrid performance when line per se selection is applied relative to the expected direct response on hybrids (formula see in Materials and Methods). Therefore, relative efficiency below one indicates that a direct selection on hybrid performance is more efficient than the indirect selection with lines per se. However, this conclusion should be interpreted cautiously since our lines and testcrosses were not always evaluated in the same environments and our results might also be affected by the lack of adaptation traits in the tropical resistance donors. In contrast, Schechert et al. (1997) observed high per se and testcross correlations of *r* = 0.94 and 0.98 for a diallel design in three locations in the US Corn Belt and recommended selection in early stages of line development. However, they still recommended to assess NCLB resistance in hybrids since the disease showed some level of heterosis (Schechert et al. 1997). On the other hand, also the inbred lines should have a minimum resistance level to ensure seed production without or with a lower number of fungicide applications.

The choice of the tester plays an important role for NCLB resistance assessment as observed in our trials. Tropical testers A and B crossed with DH lines in Brazil are known to be moderately susceptible to NCLB with a mean score of 4 (1–9 scale) (Miranda Pires, Cambé, PR, Brazil; pers. commun.). Contrarily, the flint tester C applied in Europe was highly susceptible (NCLB scoring 7–9, Kessel, Einbeck, Germany; pers. commun.) and testcrosses revealed, on average, even a higher susceptibility than the lines per se (Fig. 3). This indicates that the highly susceptible testers are recommendable for environments with low to moderate disease severities, such as in Europe.

### Brazilian genetic material is a great source of QTLs for quantitative NCLB resistance

Multi-parent QTL mapping promises to be a useful tool to dissect the genetic architecture of traits since it combines the high power to detect infrequent favorable alleles with a high mapping resolution (Würschum 2012). Connected populations are usually already available in typical breeding programs; however, each population is frequently composed by small to moderate population sizes only. As a small sample size entails a lower detection power for quantitative traits with complex/polygenic architecture (Schön et al. 2004), multi-parent QTL analysis can be an alternative to increase detection power (Han et al. 2016) in case of common QTL among families. In addition, it allows the investigation of variation in allele substitution effects, which are usually diverse at certain loci across different genetic backgrounds which increases the success rates of QTL transferability to other populations (Xu 1998; Blanc et al. 2006; Steinhoff et al. 2011; Garin et al. 2017). Hence, we conducted multi-parent QTL analysis for NCLB by means of the bi-allelic allele substitution effect models. Only donor T5 was analyzed separately in a biparental QTL mapping since it was composed by a unique family.

Our study revealed 17 QTL for NCLB_f_ on all 10 chromosomes, while each of them explained between 3.57 and 30.98% of the genotypic variance. This complex genetic architecture was also observed in other NCLB QTL studies (Wang et al. 2018; Chen et al. 2016; Van Inghelandt et al. 2012; Poland et al. 2011; Wisser et al. 2006). To the best of our knowledge, the QTLs identified in our study and located on chromosome bins 1.07, 1.08, 2.02, 2.04, 4.03, 5.04, 8.08 and 9.04 were not yet published in the literature. These findings confirm that Brazilian resistance donors are great sources of novel alleles for NCLB resistance. Although the other nine QTLs have already been identified in the same chromosome region in other studies, different genes or alleles may confer the resistance to NCLB (Chen et al. 2016; Ding et al. 2015; Schaefer and Bernardo 2013; Van Inghelandt et al. 2012; Poland et al. 2011).

In addition to minor QTL, we identified four major QTL q8, q9, q7 and q17 on chromosome bins 1.07, 2.02, 10.04 and 6.01, resp., (> 20% explained genotypic variance) originating from our Brazilian tropical donors T2 and T5. In addition to these four QTL, the QTL q4 also showed a high explained genotypic variance of 13.3 and 16.8% in Brazil and in Europe, respectively.

Among the 17 QTL identified for NCLB_f_, four originated from the adapted elite lines showing that some resistance for NCLB was already present in Europe. However, the QTL originating from the adapted elite lines explained only a lower proportion of the genotypic variance compared to most of the QTL originating from the tropical germplasm (3.57–12.27% vs. 5.43–30.98%). The five and the 14 QTLs identified in Brazilian and European trials, respectively, including two overlapping QTL, tend to be stable within Europe which were assessed in many environments composing different maize maturity growing zones and combinations of *S. turcica* races (Hanekamp 2016). In Brazil, more test locations would be necessary to confirm the stability of the identified QTL. We observed two co-located QTL between per se and testcrosses in Europe among the six QTL identified for line per se and 10 QTL identified for testcrosses (Fig. 6). This is in accordance to the moderate correlation between per se and testcrosses for NCLB_f_ in Europe (0.37 for donor T2 and 0.61 for donor T5, Fig. 4) and to the lower explained genotypic variance of testcrosses compared to lines per se for the same donor. For instance, QTL q8, which was identified for both per se and testcrosses within donor T5, had almost a twice as large allele substitution effect for line per se compared to the testcrosses. This shows that only about half of the line per se trait variance could be captured in our testcrosses which are accordance to the expectation (Melchinger et al. 1998).

The QTLs q4 and q5 for NCLB_f_ were identified in both Brazilian and European trials on bins 7.03 and 9.04, respectively. This is in line with the moderate phenotypic correlation for NCLB severity between trials in both continents (*r* = 0.36, *P* < 0.001, for donor T1, and *r* = 0.41, *P* < 0.001, for donor T2). The two QTLs can be potentially applied in breeding programs in Brazil and in Europe to assist selection of most resistant genotypes. Fine-mapping studies are, however, advisable. They can potentially increase the precision of the QTL location which is a success factor for genomics-assisted breeding application. Conducting a fine-mapping study will also show whether there is a close linkage/pleiotropy of NCLB QTL with FF QTL (q5) or just a coincidence of two different QTL in the same chromosomal segment what could be related to the large confidence interval of this FF QTL. The possibility of a pleiotropic effect cannot be discarded as the QTL q4, q7, q11, q12 and q14 where identified in the NCLB analyses of both, non-corrected and flowering time corrected data (data not shown). In addition, both QTLs were contributed by donor T2, indicating that validation studies by observing the effect of these QTL in other genetic backgrounds would be helpful before introgressing them to other genetic material.

According to the literature, the genes *Ht1, Ht2, Ht3, HtP, HtNB, Htn1* and *rt* were identified on chromosome bins 2.08, 8.06, 7.04, 2.08, 8.07, 8.05 and 3.06, respectively (for review see Galiano-Carneiro and Miedaner 2017). Except by the chromosome bin 8.05, none of our QTL were identified in these regions where the qualitative resistance genes are located indicating that our populations most likely carry quantitative resistances. Although the QTL q14 was identified on chromosome 8.05 in our populations, it is unlikely that it represents the gene *Htn1* as none of the parents were carriers of this resistance gene. In addition, the numerous QTLs attributed to NCLB resistance in our study each explaining a small to a moderate proportion of the genotypic variance only support a quantitative inheritance in our Brazilian donor lines.

### Genomics-assisted selection is a powerful breeding tool to accelerate the introgression and integration of NCLB resistance in adapted plant materials

Genomics-assisted breeding can be a good possibility to increase NCLB resistance levels in a shorter time. We investigated the applicability of these methods for our plant materials and identified high prediction accuracies for wRR-BLUP which is in accordance with studies in other pathosystems (Boeven et al. 2016; Spindel et al. 2016; Galiano-Carneiro et al. 2019; Miedaner et al. 2019; Jähne et al. 2019). RR-BLUP also presented a high prediction accuracy which is in accordance with a genomic selection (GS) assessment for NCLB resistance conducted by Technow et al. (2013) that identified prediction accuracies of 0.71 and 0.69, depending on the heterotic group. The low to moderate prediction accuracy from MAS confirms the complex genetic architecture of NCLB with many QTL with small effects only. However, accounting for epistasis can potentially increase the prediction ability as it explains a relative high proportion of the variance according to van Inghelandt et al. (2012). Conversely, the high prediction accuracies in this cross-validation study may be overestimated since our training and prediction sets were composed by closely related plant materials and were tested in the same environments, both can considerably inflate the estimates (Riedelsheimer et al. 2013; Brauner et al. 2020). The prediction accuracies for GS from tests in Brazil were lower than those from Europe, most probably due to the lower testing intensity in Brazil. Many other factors can also influence the prediction accuracy such as training set size and relationship between the training and prediction sets. We tested different training set sizes and observed a linear increase of the prediction accuracy as we increased the number of individuals within the training set (data not shown). This result is in accordance with other studies (Riedelsheimer et al. 2013; Han et al. 2016; Van Inghelandt et al. 2019). For this reason, we compared at a fixed training set of 60 individuals the prediction accuracies of materials with different genetic relationships between the training and prediction sets (Fig. 9). An increase in relatedness between training and prediction sets increased the prediction ability (Riedelsheimer et al. 2013; Han et al. 2018; Brauner et al. 2020). The lowest prediction ability was observed for predictions between heterotic pools (i.e., between T1 and T2) which is in accordance with other studies related to different traits (Han et al. 2018, Brauner et al. 2020). Van Inghelandt et al. (2019) observed an increase of the prediction ability when a mix of individuals from different pools were composing the training and prediction sets. In this study, we followed a similar approach when using populations of T1 and T2 in the training set to predict a population of the opposite donor, but this did not increase the prediction accuracy (Fig. 9). This could be due to the lower diversity of the mixed populations included in our work compared to Van Inghelandt et al. (2019)’s populations.

One aspect that can hamper the application of the identified QTLs in breeding programs in Europe is the lack of adaptation traits of the Brazilian germplasm, especially when traits unwanted for European conditions, such as late maturity, photoperiod sensitivity and plant tallness, are located in close genomic regions to our identified QTL. The three major NCLB QTLs that could be recommended for introgression due to their high explained genetic variance and stability (e.g., q4, q8 and q9) were not linked to the QTL identified for FF and PH. Therefore, concomitant selection to reduce NCLB damage, early maturity and short plant height is feasible.

## Conclusions

Quantitative resistances tend to be the best option to keep low NCLB levels durably in areas with high disease pressure. This type of resistance is especially important for NCLB resistance because *S. turcica* populations have a moderate to high evolutionary potential (McDonald and Linde 2002) leading to vulnerability of race-specific resistances. Minor and major QTL were identified for NCLB in our study explaining 3.57–30.98% of the genetic variance. Among them, two QTL were detected in Brazil and Europe explaining between 7.10 and 16.83% of the genotypic variance, which can be employed in a broad range of ecosystems.

Brazilian breeding materials were quantitatively resistant in all our European test locations and the crosses between Brazilian × European lines yielded moderate to high genetic variances for NCLB resistance. However, other resistance sources from Brazil can potentially also result in stable quantitative resistance to NCLB with even higher resistance levels. Therefore, we recommend further investigations on South American donor lines.

Before the application of these environmentally highly stable two QTLs in genomics-assisted breeding programs, QTL validation in different BC populations is recommended. This should improve the precision of the QTL location and the success rates of QTL transferability by molecular markers. For this, KASP (Kompetitive allele specific PCR) markers based on the sequences of the detected closely linked SNPs for foreground selection that allow a low-cost detection in segregating backcross generations should be generated. Finally, a genomics-assisted breeding approach can be applied for a successful introgression and integration of NCLB resistance QTL originating from tropical plant material.

## Electronic supplementary material

Below is the link to the electronic supplementary material.

Supplementary material 1 (PDF 247 kb)

Supplementary material 2 (PDF 650 kb)

## References

[CR1] Agrarmeteorologie Baden-Württemberg (2020) Tagesmittelwerte des Monats Wetterstation Stutensee. https://www.wetter-bw.de/Internet/AM/inetcntrBW.nsf/cuhome.xsp?src=GSSGT0B084&p1=title%3DStutensee%7E%7Eurl%3D%2FInternet%2FAM%2FNotesBwAM.nsf%2FXP_ABC_All%2F2FC0D2991AD1DC2FC1257FBC00411C21%3FOpenDocument&p3=1H58NLY654&p4=EZ5D5ZTI3K Accessed 28 Jan 2020

[CR2] AgrarMeteorologie Bayer (2020) Tagesmittelwerte des Monats Wetterstation Bärnau. https://www.am.rlp.de/Internet/AM/NotesBAM.nsf/bamweb/439533daa65b8ba4c1257393002d90c5?OpenDocument&TableRow=3.1.1%2C3.4#3.1 Accessed 28 Jan 2020

[CR3] Balint-Kurti PJ, Yang J, Van Esbroeck G (2010). Use of a maize advanced intercross line for mapping of QTL for Northern leaf blight resistance and multiple disease resistance. Crop Sci.

[CR4] Bergquist RR, Masias OR (1974). Physiologic specialization in *Trichometasphaeria turcica* f. sp. *zeae* and *T. turcica* f. sp. sorghi in Hawaii. Phytopathology.

[CR5] Bernal-Vasquez AM, Utz HF, Piepho HP (2016). Outlier detection methods for generalized lattices: a case study on the transition from ANOVA to REML. Theor Appl Genet.

[CR6] Bernardo R (2014). Genomewide selection when major genes are known. Crop Sci.

[CR7] Blanc G, Charcosset A, Mangin B (2006). Connected populations for detecting quantitative trait loci and testing for epistasis: an application in maize. Theor Appl Genet.

[CR8] Boeven PHG, Longin CFH, Leiser WL (2016). Genetic architecture of male floral traits required for hybrid wheat breeding. Theor Appl Genet.

[CR9] Bormann CA, Rickert AM, Ruiz RA (2004). Tagging quantitative trait loci for maturity-corrected late blight resistance in tetraploid potato with PCR-based candidate gene markers. Mol Plant Microbe Interact.

[CR10] Brauner PC, Müller D, Molenaar WS, Melchinger AE (2020). Genomic prediction with multiple biparental families. Theor Appl Genet.

[CR11] Broman KW, Sen Ś (2009). A guide to QTL mapping with R/qtl.

[CR12] Broman KW, Wu H, Sen Ś, Churchill GA (2003). R/qtl: QTL mapping in experimental crosses. Bioinformatics.

[CR13] Bundessortenamt Sortenliste (2019) https://www.bundessortenamt.de/bsa/media/Files/BSL/bsl_getreide_2019.pdf Accessed 16 Jan 2020

[CR14] Carson ML (1999). “Helminthosporium” leaf spots and blights, in compendium of corn diseases, herausgeber: white DG.

[CR15] Chen G, Wang X, Long S (2016). Mapping of QTL conferring resistance to Northern corn leaf blight using high-density SNPs in maize. Mol Breed.

[CR16] CONAB (2020) Boletim da Safra de Grãos – Companhia Nacional de Abastecimento (Conab) https://www.conab.gov.br/info-agro/safras/graos/boletim-da-safra-de-graos Accessed 17 Jan 2020

[CR17] Cooper M, Delacy IH, Basford KE, Hammer GL (1994). Relationships among analytical methods used to analyse genotypic adaptation in multi-environment trials. Theor Appl Genet.

[CR18] Cramptom BG (2015) Northern corn leaf blight in maize and sorghum—piecing together the puzzle. Dupont Plant Breeding Symposium 2015, Pretoria, 29 September

[CR19] Cullis BR, Smith AB, Coombes NE (2006). On the design of early generation variety trials with correlated data. J Agric Biol Environ Stat.

[CR20] Czarnak-Kłos M, Rodríguez-Cerezo E (2010) Best practice documents for coexistence of genetically modified crops with conventional and organic farming: Maize crop production. European coexistence bureau (ECoB). https://ec.europa.eu/jrc/sites/jrcsh/files/ecob_best_practice_maize.pdf. Accessed 9 Feb 2020

[CR21] De Rossi R, Plazas M, Brucher E, Ducasse D, Guerra G (2010) El Tizón del Maíz (*Exserohilum turcicum*): presencia e impacto en el centro norte de Córdoba durante tres campañas agrícolas [The North corn leaf blight (*Exserohilum turcicum*): presence and impact in the center north of Córdoba during three growing seasons, translation from Spanish]. Actas IX Congreso Nacional de Maíz, Rosario, Argentina, 17–19 November

[CR22] Ding J, Ali F, Chen G (2015). Genome-wide association mapping reveals novel sources of resistance to northern corn leaf blight in maize. BMC Plant Biol.

[CR23] Emrich K, Wilde F, Miedaner T, Piepho HP (2008). REML approach for adjusting the Fusarium head blight rating to a phenological date in inoculated selection experiments of wheat. Theor Appl Genet.

[CR24] Endelman JB (2011). Ridge regression and other kernels for genomic selection with R package rrBLUP. Plant Genome J.

[CR25] Endelman JB, Jannink J (2012). Shrinkage estimation of the realized relationship matrix. G3 Genes Genomes Genet.

[CR26] Falconer DS, Mackay TFC (1996). Introduction to quantitative genetics.

[CR27] Gaikpa DS, Koch S, Fromme FJ (2020). Genome-wide association mapping and genomic prediction of Fusarium head blight resistance, heading stage and plant height in winter rye (*Secale cereale*). Plant Breed.

[CR28] Galiano-Carneiro AL, Miedaner T (2017). Genetics of resistance and pathogenicity in the maize/*Setosphaeria turcica* pathosystem and implications for breeding. Front Plant Sci.

[CR29] Galiano-Carneiro AL, Boeven PHG, Maurer HP (2019). Genome-wide association study for an efficient selection of *Fusarium* head blight resistance in winter triticale. Euphytica.

[CR30] Ganal MW, Durstewitz G, Polley A (2011). A large maize (*Zea mays* L.) SNP genotyping array: development and germplasm genotyping, and genetic mapping to compare with the B73 reference genome. PLoS ONE.

[CR31] Garin V, Wimmer V, Mezmouk S (2017). How do the type of QTL effect and the form of the residual term influence QTL detection in multi-parent populations? A case study in the maize EU-NAM population. Theor Appl Genet.

[CR32] Garin V, Wimmer V, Borchardt D, Malosetti M, van Eeuwijk F (2018) mppR: multi-parent population QTL analysis. R package version 1.2.0

[CR33] Gilmour AR, Gogel BJ, Cullis BR, Thompson R (2009). ASReml user guide release 3.0.

[CR34] Haley CS, Knott SA (1992). A simple regression method for mapping quantitative trait loci in line crosses using flanking markers. Heredity.

[CR35] Han S, Utz HF, Liu W (2016). Choice of models for QTL mapping with multiple families and design of the training set for prediction of Fusarium resistance traits in maize. Theor Appl Genet.

[CR36] Han S, Miedaner T, Utz HF (2018). Genomic prediction and GWAS of Gibberella ear rot resistance traits in dent and flint lines of a public maize breeding program. Euphytica.

[CR37] Hanekamp H (2016) Europäisches Rassen-Monitoring und Pathogenesestudien zur Turcicum-Blattdürre (*Exserohilum turcicum*) an Mais (*Zea mays* L.). [European race monitoring and pathogenesis studies for Northern corn leaf blight (*Exserohilum turcicum*) in maize, translation from German]. Ph.D. study, University of Göttingen, Germany

[CR39] Herter CP, Ebmeyer E, Kollers S (2019). An experimental approach for estimating the genomic selection advantage for *Fusarium* head blight and *Septoria tritici* blotch in winter wheat. Theor Appl Genet.

[CR40] Hooker AL (1973) Northern leaf blight. In: Nelson RR (ed) Breeding plants for disease resistance. The Pennsylvania State University, State College, PA, pp 135–137

[CR41] Hurni S, Scheuermann D, Krattinger SG (2015). The maize disease resistance gene *Htn1* against Northern corn leaf blight encodes a wall-associated receptor-like kinase. Proc Natl Acad Sci.

[CR42] Ilmeteo (2020) Archivio meteo Rivignano https://www.ilmeteo.it/portale/archivio-meteo/Rivignano. Accessed 18 Jan 2020

[CR43] INMET (2020) Instituto nacional de meteorologia. http://www.inmet.gov.br/portal/index.php?r=clima/normaisClimatologicas. Accessed 28 Jully 2020

[CR45] Jähne F, Balko C, Hahn V (2019). Cold stress tolerance of soybeans during flowering: QTL mapping and efficient selection strategies under controlled conditions. Plant Breed.

[CR46] Jiang C, Edmeades GO, Armstead I, Lafitte HR, Hayward MD, Hoisington D (1999). Genetic analysis of adaptation differences between highland and lowland tropical maize using molecular markers. Theor Appl Genet.

[CR47] Kaefer KAC, Schuelter AR, Schuster I, Marcolin J, Vendrusco ECG (2017). Association mapping and genetic control for Northern leaf blight (*Exserohilum turcicum*) resistance in maize lines. Aust J Crop Sci.

[CR48] Löffler M, Kessel B, Ouzunova M, Miedaner T (2011). Covariation between line and testcross performance for reduced mycotoxin concentrations in European maize after silk channel inoculation of two Fusarium species. Theor Appl Genet.

[CR49] Ma Z, Liu B, He S, Gao Z (2020). Analysis of physiological races and genetic diversity of *Setosphaeria turcica* (Luttr.) K.J. Leonard Suggs from different regions of China. Can J Plant Path.

[CR50] Maize GBD (2020) Maize genetics and genomics database. https://www.maizegdb.org/gene_center/gene#gm_downloads. Accessed 3 Feb 2020

[CR52] Mayer M, Unterseer S, Bauer E (2017). Is there an optimum level of diversity in utilization of genetic resources?. Theor Appl Genet.

[CR53] McDonald BA, Linde C (2002). Pathogen population genetics, evolutionary potential, and durable resistance. Annu Rev Phytopathol.

[CR54] Melchinger AE, Utz HF, Schön CC (1998). Quantitative trait locus (QTL) mapping using different testers and independent population samples in maize reveals low power of QTL detection and large bias in estimates of QTL effects. Genetics.

[CR55] Meteociel (2020) Données mensuelles pour Mont-de-Marsan https://www.meteociel.fr/climatologie/villes.php?code=7607&mois=9&annee=2019 Accessed 28 Jan 2020

[CR56] Miedaner T, Rapp M, Flath K (2019). Genetic architecture of yellow and stem rust resistance in a durum wheat diversity panel. Euphytica.

[CR57] Money D, Gardner K, Migicovsky Z (2015). LinkImpute: fast and accurate genotype imputation for non-model organisms. Genes Genomes Genet.

[CR58] Nwanosike MR, Mabagala RB, Kusolwa PM (2015). Disease intensity and distribution of *Exserohilum turcicum* incitant of Northern leaf blight of maize in Tanzania. Int J Pure Appl Bioscie.

[CR59] OECD/FAO (2019) OECD–FAO Agricultural Outlook 2019–2028, OECD Publishing, Paris/Food and Agriculture Organization of the United Nations, Rome. 10.1787/agr_outlook-2019-en

[CR60] Piepho HP, Büchse A, Truberg B (2006). On the use of multiple lattice designs and α-designs in plant breeding trials. Plant Breed.

[CR61] Piepho HP, Williams ER, Fleck M (2006). A note on the analysis of designed experiments with complex treatment structure. HortScience.

[CR62] Pilet-Nayel M-L, Moury B, Caffier V (2017). Quantitative resistance to plant pathogens in pyramiding strategies for durable crop protection. Front Plant Sci.

[CR63] Poland JA, Bradbury PJ, Buckler ES, Nelson RJ (2011). Genome-wide nested association mapping of quantitative resistance to Northern leaf blight in maize. Proc Natl Acad Sci USA.

[CR76] R Core Team (2018) R: a language and environment for statistical computing. R Foundation for Statistical Computing, Vienna, Austria. https://www.R-project.org/

[CR64] Raymundo A, Hooker A (1981). Measuring the relationship between Northern corn leaf blight and yield losses. Plant Diseases.

[CR65] Ribeiro RM, Do Amaral Júnior AT, Pena GF (2016). Histórico da helmintosporiose em sete ciclos de seleção recorrente na população UENF-14 de milho-pipoca. Acta Sci Agron.

[CR66] Riedelsheimer C, Endelman JB, Stange M (2013). Genomic predictability of interconnected biparental maize populations. Genetics.

[CR67] Romero LR (2016) Occurrence and importance of foliar diseases on maize (*Zea mays* L.) in Central Europe. Ph.D. study, University of Göttingen, Germany

[CR68] Rüdelsheim PLJ, Smets G (2011) Baseline information on agricultural practices in the EU Maize (*Zea mays* L.). https://www.europabio.org/sites/default/files/120702_report_eu_farming_practices_maize.pdf Accessed 9 Feb 2020

[CR69] Sánchez-Martín J, Keller B (2019). Contribution of recent technological advances to future resistance breeding. Theor Appl Genet.

[CR70] Schaefer CM, Bernardo R (2013). Genomewide association mapping of flowering time, kernel composition, and disease resistance in historical Minnesota maize inbreds. Crop Sci.

[CR71] Schechert A, Geiger HH, Welz HG (1997) Generation means and combining ability analysis of resistance to *Setosphaeria turcica* in African maize. Maize productivity gains through research and technology dissemination. In: Ransom JK, Palmer AFE, Zambezi BT, Mduruma ZO, Waddington SR, Pixley KV, et al. Proceedings of the fifth eastern and southern africa regional maize conference, (Arusha: CIMMYT), pp 212–218

[CR72] Schön CC, Utz HF, Groh S (2004). Quantitative trait locus mapping based on resampling in a vast maize testcross experiment and its relevance to quantitative genetics for complex traits. Genetics.

[CR73] Spindel JE, Begum H, Akdemir D (2016). Genome-wide prediction models that incorporate de novo GWAS are a powerful new tool for tropical rice improvement. Heredity.

[CR74] Steinhoff J, Liu W, Maurer HP (2011). Multiple-line cross quantitative trait locus mapping in European elite maize. Crop Sci.

[CR75] Stram DO, Lee JW (1994). Variance components testing in the longitudinal mixed effects model. Biometrics.

[CR77] Technow F, Bürger A, Melchinger AE (2013). Genomic prediction of northern corn leaf blight resistance in maize with combined or separated training sets for heterotic groups. G. Genes Genomes Genetics.

[CR78] Tefferi A, Hulluka M, Welz HG (1996). Assessment of damage and grain yield loss in maize caused by Northern leaf blight in western Ethiopia. J Plant Dis Prot.

[CR79] Time and date (2020) Past weather in Graz, Styria, Austria. https://www.timeanddate.com/weather/austria/graz/historic?month=9&year=2019. Accessed 3 Jan 2020

[CR80] USDA/IPAD (2020) World Agricultural Production U.S. Department of Agriculture Foreign Agricultural Service/Office of Global Analysis International Production Assessment Division (IPAD). https://apps.fas.usda.gov/psdonline/circulars/production.pdf. Accessed 14 Jan 2020

[CR81] Utz HF, Melchinger AE, Schön CC (2000). Bias and sampling error of the estimated proportion of genotypic variance explained by quantitative trait loci determined from experimental data in maize using cross validation and validation with independent samples. Genetics.

[CR82] Van Inghelandt D, Melchinger AE, Martinant JP, Stich B (2012). Genome-wide association mapping of flowering time and northern corn leaf blight (*Setosphaeria turcica*) resistance in a vast commercial maize germplasm set. BMC Plant Biol.

[CR83] Van Inghelandt D, Frey FP, Ries D (2019). QTL mapping and genome-wide prediction of heat tolerance in multiple connected populations of temperate maize. Sci Rep.

[CR84] Wang J, Xu Z, Yang J (2018). qNCLB7.02, a novel QTL for resistance to Northern corn leaf blight in maize. Mol Breed.

[CR85] Welz HG (1998). Genetics and epidemiology of the pathosystem *Zea mays*/*Setosphaeria turcica*.

[CR86] Welz HG, Bassetti P, Geiger HH (1996). Turcicum-Blattdürre und Aleppohirse: zwei Schaderreger auf dem Vormarsch [Northern corn leaf blight and Aleppo millet: two pests on the rise, translation from German]. Mais.

[CR87] Welz HG, Xia XC, Bassetti P (1999). QTLs for resistance to *Setosphaeria turcica* in an early maturing Dent × Flint maize population. Theor Appl Genet.

[CR88] Whittaker JC, Thompson R, Denham MC (2000). Marker-assisted selection using ridge regression. Genet Res.

[CR89] Wisser RJ, Balint-Kurti PJ, Nelson RJ (2006). The genetic architecture of disease resistance in maize: a synthesis of published studies. Phytopathology.

[CR90] Würschum T (2012). Mapping QTL for agronomic traits in breeding populations. Theor Appl Genet.

[CR91] Würschum T, Liu W, Gowda M (2012). Comparison of biometrical models for joint linkage association mapping. Heredity.

[CR92] Xu S (1998). Mapping quantitative trait loci using multiple families of line crosses. Genetics.

[CR93] Yu J, Pressoir G, Briggs WH (2006). A unified mixed-model method for association mapping that accounts for multiple levels of relatedness. Nat Genet.

[CR94] Zeng ZB (1993). Theoretical basis for separation of multiple linked gene effects in mapping quantitative trait loci. Proc Natl Acad Sci U S A.

[CR95] Zeng ZB (1994). Precision mapping of quantitative trait loci. Genetics.

[CR96] Zhao Y, Mette MF, Gowda M (2014). Bridging the gap between marker-assisted and genomic selection of heading time and plant height in hybrid wheat. Heredity.

