# Supplementary material for: Intercontinental trials reveal stable QTL for Northern corn leaf blight resistance in Europe and in Brazil
Source: Theor Appl Genet. 2020 Sep 30;134(1):63–79. doi: 10.1007/s00122-020-03682-1 (PMC7813747; doi:10.1007/s00122-020-03682-1)
Supplement: Supplementary file 1 — Supplementary material 1 (PDF 247 kb) [file 122_2020_3682_MOESM1_ESM.pdf]

Supplementary material

Intercontinental trials reveal stable QTL for Northern corn leaf blight resistance in Europe and in Brazil

Ana L. Galiano-Carneiro[1], Bettina Kessel[2], Thomas Presterl[2] and Thomas Miedaner[1]✉

[1]State Plant Breeding Institute, University of Hohenheim, Stuttgart, Germany

[2]Kleinwanzlebener Saatzucht (KWS) KWS SAAT SE & Co. KGaA, Einbeck, Germany

✉ Corresponding author: miedaner@uni-hohenheim.de

ORCID ID:

Ana L. Galiano-Carneiro: https://orcid.org/0000-0002-2615-3510

Thomas Miedaner: *https://orcid.org/0000-0002-9541-3726*

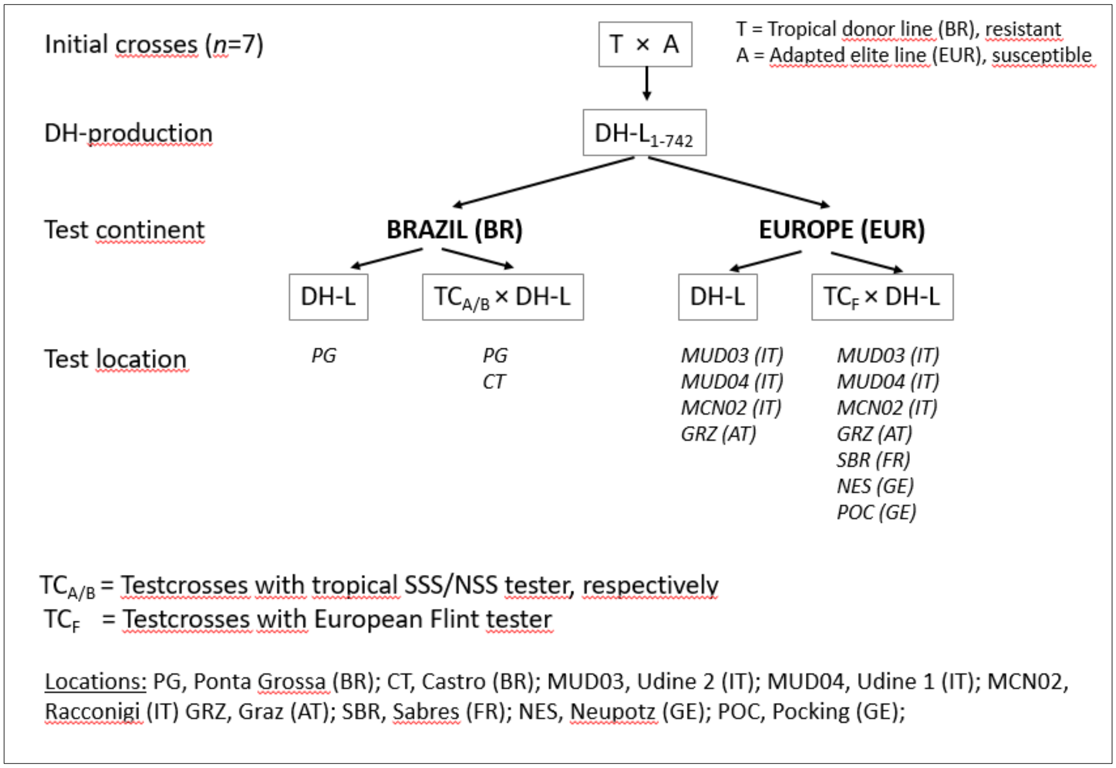

Supplementary Fig. 1 Schematic illustration of the genetic materials and testing locations

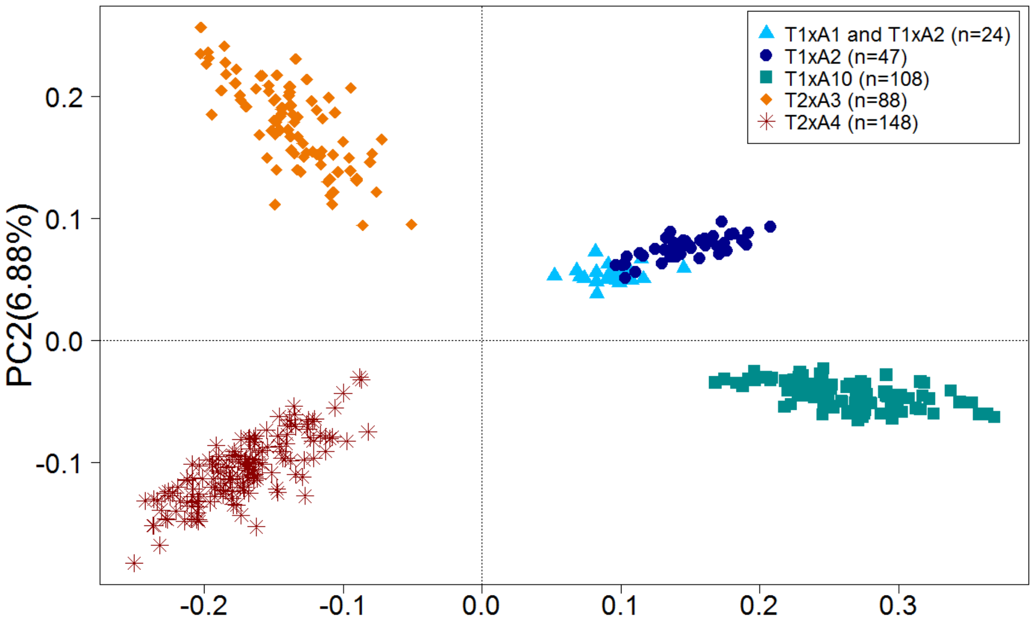

Supplementary Fig. 2 Principal component (PC) analysis based on modified Rogger's distance for populations T1×A1, T1×A2, T1×A10, T2×A3 and T2×A4, where the prefixes "T" and "A" are assigned to "tropical" and "adapted" double haploid (DH) parents, respectively. Populations T2×A5 and T5×A11 were not included in the PC due to the low number of overlapping markers with the presented population

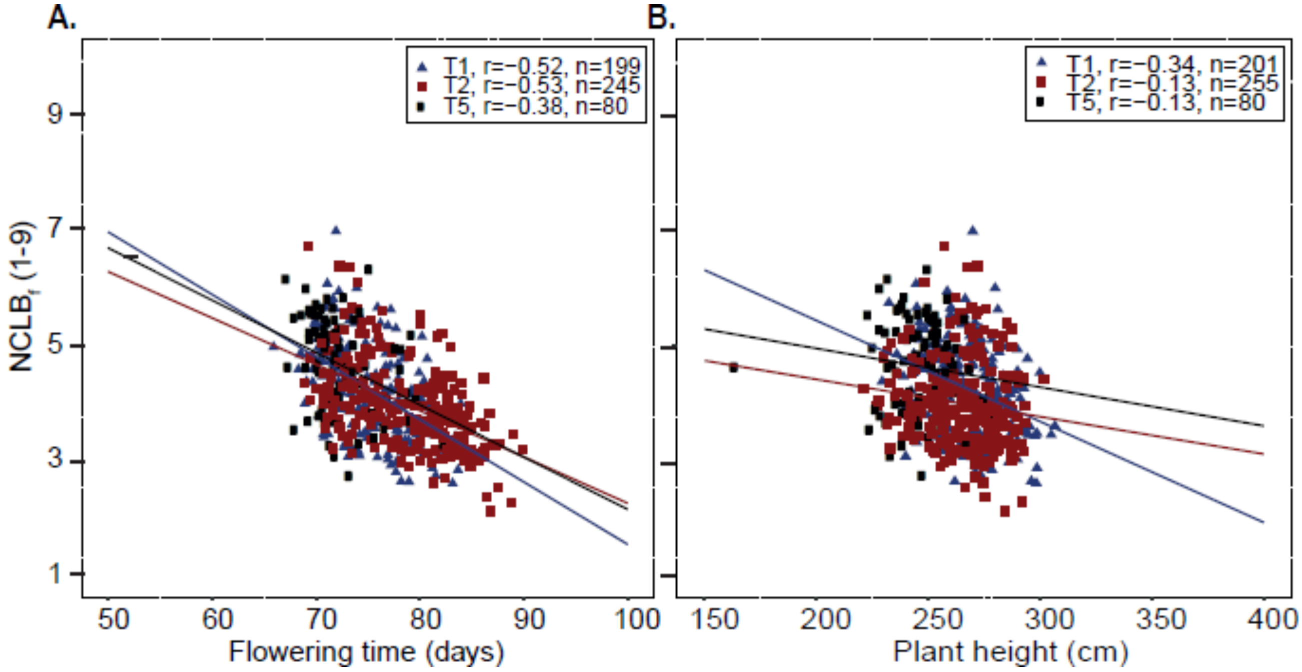

Supplementary Fig. 3 Scatter plots for final NCLB score (NCLB$_f$) and flowering time evaluated as testcrosses in Europe (a) and NCLB$_f$ plotted against plant height evaluated as testcrosses in Europe (b) as well as the phenotypic correlation (r) and number of genotypes (n)
