# Supplementary material for: Intercontinental trials reveal stable QTL for Northern corn leaf blight resistance in Europe and in Brazil
Source: Theor Appl Genet. 2020 Sep 30;134(1):63–79. doi: 10.1007/s00122-020-03682-1 (PMC7813747; doi:10.1007/s00122-020-03682-1)
Supplement: Supplementary file 2 — Supplementary material 2 (PDF 650 kb) [file 122_2020_3682_MOESM2_ESM.pdf]

Supplementary material

Intercontinental trials reveal stable QTL for Northern corn leaf blight resistance in Europe and in Brazil

Ana L. Galiano-Carneiro[1], Bettina Kessel[2], Thomas Presterl[2] and Thomas Miedaner[1]✉

[1]State Plant Breeding Institute, University of Hohenheim, Stuttgart, Germany

[2]Kleinwanzlebener Saatzucht (KWS) KWS SAAT SE & Co. KGaA, Einbeck, Germany

✉ Corresponding author: miedaner@uni-hohenheim.de

ORCID ID:

Ana L. Galiano-Carneiro: https://orcid.org/0000-0002-2615-3510

Thomas Miedaner: https://orcid.org/0000-0002-9541-3726

Supplementary Table 1 Environments where Northern corn leaf blight (NCLB), female flowering time (FF) and plant height (PH) were assessed and the respective population size (n) of the crosses. The traits were assessed in a total of seven locations in Europe and two in Brazil. The years when each location was assessed is described in brackets

Pop	n	NCLB		FF, PH
		Brazil	Europe	Europe
Line per se				
T1×A1	31	PG (19)	-	-
T1×A2	49	PG (19)	-	-
T1×A10	11	PG (19)	-	-
T2×A3	102	PG (19)	MUD04 (17)	-
T2×A4	162	PG (19)	MUD04 (17)	-
T2×A5	112	-	MUD03, MUD04 (18); MUD04, GRZ, MCN02 (19)	VIA (18, 19)
T5×A11	130	-	MUD03 (18); MUD04, MCN02 (19)	VIA (18, 19)
Test cross				
T1×A1	35	CT, PG (19)	MUD03, MUD04, POC, SBR (18); MUD04, POC, SBR, NES, GRZ, MCN02 (19)	VIA, NES (18); VIA (19)
T1×A2	60	CT, PG (19)	MUD03, MUD04, POC, SBR (18); MUD04, POC, SBR, NES, GRZ, MCN02 (19)	VIA, NES (18); VIA (19)
T1×A10	114	CT, PG (19)	MUD03, MUD04, POC, SBR (18); MUD04, POC, SBR, NES, GRZ, MCN02 (19)	VIA, NES (18); VIA (19)
T2×A3	102	CT, PG (19)	MUD03, MUD04, POC (18); MUD04, POC, SBR, NES, GRZ, MCN02 (19)	VIA, NES (18); VIA (19)
T2×A4	162	CT, PG (19)	MUD03, MUD04, POC (18); MUD04, POC, SBR, NES, GRZ, MCN02 (19)	VIA, NES (18); VIA (19)
T2×A5	93	-	MUD04 (18); NES, GRZ, MCN02 (19)	VIA, NES (18); VIA (19)
T5×A11	102	-	MUD04 (18); MUD04; NES, MCN02 (19)	VIA, NES (18); VIA (19)

PG, Ponta Grossa; CT, Castro; MUD04, Udine 1 (IT); MUD03, Udine 2 (IT); POC, Pocking (GE); SBR, Sabres (FR); NES, Neupotz (GE); GRZ, Graz (AT); MCN02, Racconigi (IT); VIA, Vicenza – Battistella (IT)

Supplementary Table 2 Climatic information for each testing environment. Sowing (Sow.), inoculation (Inoc.) and scoring (S1-4) dates to each trial, region, type of plant material (line *per se*, PS, Testcross, TC), numbers of days from scoring to inoculation (days post inoculation, dpi) in addition to the mean (T_{av}), minimum (T_{min}) and maximum (T_{max}) temperatures and relative humidity (H) from inoculation date to last scoring are indicated

Trials	Region	Type	Location	Sow.	Inoc.	S1	S2	S3	S4	dpi1	dpi2	dpi3	dpi4	T_{av} (°C)	T_{min} (°C)	T_{max} (°C)	H (%)	Weather source
2017																		
2	Europe	PS	MUD04	20.4	27.6	23.8	7.9	-	-	57	72	-	-	24.0	17.3	29.9	64.8	Ilmeteo 2020
2018																		
3	Europe	PS	MUD03	11.5	5.7	22.8	1.9	7.9	14.9	48	58	64	71	24.2	18.4	30.3	68.3	Ilmeteo 2020
4	Europe	PS	MUD03	11.5	5.7	22.8	31.8	7.9	14.9	48	57	-	-	24.2	18.4	30.3	68.3	Ilmeteo 2020
4	Europe	PS	MUD04	3.5	5.7	21.8	1.9	7.9	13.9	47	58	64	70	24.2	18.4	30.3	68.3	Ilmeteo 2020
1	Europe	TC	MUD03	11.5	5.7	22.8	1.9	7.9	-	48	58	64	-	24.2	18.4	30.3	68.3	Ilmeteo 2020
2	Europe	TC	MUD03	11.5	5.7	2.9	7.9	14.9	-	59	64	71	-	24.2	18.4	30.3	68.3	Ilmeteo 2020
1	Europe	TC	MUD04	3.5	5.7	21.8	1.9	7.9	-	47	58	64	-	24.2	18.4	30.3	68.3	Ilmeteo 2020
2	Europe	TC	MUD04	3.5	5.7	21.8	1.9	7.9	13.9	47	58	64	70	24.2	18.4	30.3	68.3	Ilmeteo 2020
3	Europe	TC	MUD04	3.5	5.7	21.8	1.9	7.9	-	47	58	64	-	24.2	18.4	30.3	68.3	Ilmeteo 2020
4	Europe	TC	MUD04	3.5	5.7	21.8	1.9	7.9	-	47	58	64	-	24.2	18.4	30.3	68.3	Ilmeteo 2020
1	Europe	TC	POC	5.5	21.6	17.8	26.8	5.9	12.9	57	66	76	83	19.6	7.4	26.3	78.8	AgrarMeteorologie Bayer 2020
2	Europe	TC	POC	5.5	30.6	17.8	26.8	5.9	12.9	48	57	67	74	19.6	7.4	26.3	78.8	AgrarMeteorologie Bayer 2020
1	Europe	TC	SBR	4.5	21.6	27.8	5.9	-	-	67	76	-	-	29.0	6.7	37.0	-	Meteociel 2020
2019																		
1	Brazil	PS	PG	5.11	8.1	1.3	9.3	14.3	-	52	60	65	-	20.4	17.5	27.8	-	Inmet 2020
2	Brazil	PS	PG	5.11	8.1	1.3	14.3	-	-	52	65	-	-	20.4	17.5	27.8	-	Inmet 2020
4	Europe	PS	MUD04	7.6	1.8	28.8	10.9	20.9	1.10	27	40	50	61	23.5	17.7	29.1	-	Ilmeteo 2020
3	Europe	PS	MUD04	7.6	1.8	28.8	10.9	20.9	1.10	27	40	50	61	23.5	17.7	29.1	-	Ilmeteo 2020
4	Europe	PS	MCN02	10.5	11.7	22.8	3.9	10.9	-	61	54	42	-	23.2	17.1	27.1	69.1	Ilmeteo 2020
3	Europe	PS	MCN02	10.5	11.7	22.8	3.9	10.9	-	61	54	42	-	23.2	17.1	27.1	69.1	Ilmeteo 2020
4	Europe	PS	GRZ	16.4	1.7	6.9	11.9	19.9	30.9	67	72	80	91	19.3	2.0	33.0	72.0	Time and date 2020
1	Brazil	TC	CT	19.1	-	28.2	7.3	15.3	-	-	-	-	-	20.4	17.5	27.8	-	Inmet 2020
1	Brazil	TC	PG	6.1	8.1	1.3	11.3	14.3	-	52	62	65	-	20.4	17.5	27.8	-	Inmet 2020
2	Brazil	TC	CT	19.1	-	28.2	7.3	15.3	-	-	-	-	-	20.4	17.5	27.8	-	Inmet 2020
2	Brazil	TC	PG	6.1	8.1	1.3	10.3	15.3	-	52	61	66	-	20.4	17.5	27.8	-	Inmet 2020
1	Europe	TC	MUD04	7.6	1.8	28.8	10.9	20.9	1.10	27	40	50	61	23.5	17.7	29.1	-	Ilmeteo 2020
2	Europe	TC	MUD04	7.6	1.8	28.8	10.9	20.9	1.10	27	40	50	61	23.5	17.7	29.1	-	Ilmeteo 2020
3	Europe	TC	MUD04	7.6	1.8	28.8	10.9	20.9	1.10	27	40	50	61	23.5	17.7	29.1	-	Ilmeteo 2020
1	Europe	TC	MCN02	10.5	11.7	22.8	3.9	10.9	-	61	54	42	-	23.2	17.1	27.1	69.1	Ilmeteo 2020
2	Europe	TC	MCN02	10.5	11.7	22.8	3.9	10.9	-	61	54	42	-	23.2	17.1	27.1	69.1	Ilmeteo 2020

Supplementary Table 2 (continued)

Trials	Region	Type	Location	Sow.	Inoc.	S1	S2	S3	S4	dpi1	dpi2	dpi3	dpi4	T_{av} (°C)	T_{min} (°C)	T_{max} (°C)	H (%)	Weather source
4	Europe	TC	MCN02	10.5	11.7	22.8	3.9	10.9	-	61	54	42	-	23.2	17.1	27.1	69.1	Ilmeteo 2020
3	Europe	TC	MCN02	10.5	11.7	22.8	3.9	10.9	-	61	54	42	-	23.2	17.1	27.1	69.1	Ilmeteo 2020
1	Europe	TC	POC	7.5	27.6	28.8	6.9	13.9	20.9	62	71	78	85	18.6	8.6	26.0	83.9	AgrarMeteorologie Bayer 2020
2	Europe	TC	POC	7.5	4.7	28.8	6.9	13.9	20.9	55	64	71	78	18.6	8.6	26.0	83.9	AgrarMeteorologie Bayer 2020
1	Europe	TC	NES	1.5	9.7	3.9	12.9	20.9	-	56	65	73	-	19.6	15.3	20.8	72.1	Agrarmeteorologie Baden-Württemberg 2020
2	Europe	TC	NES	1.5	9.7	3.9	12.9	20.9	-	56	65	73	-	19.6	15.3	20.8	72.1	Agrarmeteorologie Baden-Württemberg 2020
4	Europe	TC	NES	1.5	9.7	3.9	12.9	20.9	-	56	65	73	-	19.6	15.3	20.8	72.1	Agrarmeteorologie Baden-Württemberg 2020
3	Europe	TC	NES	1.5	9.7	3.9	12.9	20.9	-	56	65	73	-	19.6	15.3	20.8	72.1	Agrarmeteorologie Baden-Württemberg 2020
1	Europe	TC	SBR	30.4	20.6	20.8	27.8	5.9	12.9	61	68	77	84	28.2	3.7	38.4	-	Meteociel 2020
2	Europe	TC	SBR	30.4	11.7	20.8	27.8	5.9	12.9	40	47	56	63	28.2	3.7	38.4	-	Meteociel 2020
1	Europe	TC	GRZ	16.4	1.7	6.9	11.9	19.9	30.9	67	72	80	91	19.3	2.0	33.0	72.0	Time and date 2020
2	Europe	TC	GRZ	16.4	1.7	6.9	11.9	19.9	30.9	67	72	80	91	19.3	2.0	33.0	72.0	Time and date 2020
4	Europe	TC	GRZ	16.4	1.7	6.9	11.9	19.9	30.9	67	72	80	91	19.3	2.0	33.0	72.0	Time and date 2020

Supplementary Table 3a Statistics summary and variance components for Northern corn leaf blight mean ($NCLB_m$) and final score ($NCLB_f$) in Brazil. The table includes the minimum (Min), median, mean, maximum (Max.) BLUEs for each family within a trial. Number of genotypes (n), least square of a difference (LSD), genetic variance (σ_G^2), genotype and location interactions ($\sigma_{G\times L}^2$), residuals (σ_e^2) and heritability according to Cullis et al. (2006) (H^2) to each population

**Brazil**	**NCLBm (1-9)**					**NCLBf (1-9)**				
	**Trial 1**			**Trial 2**		**Trial 1**			**Trial 2**	
	**T1xA1**	**T1xA2**	**T1xA10**	**T2xA3**	**T2xA4**	**T1xA1**	**T1xA2**	**T1xA10**	**T2xA3**	**T2xA4**
Per se										
Min	2.21	1.95	2.42	0.92	0.92	2.52	2.37	3.52	1.35	1.35
Median	4.49	4.82	5.38	2.75	2.55	5.32	5.47	5.60	3.36	3.22
Mean	4.32	4.93	5.44	3.22	3.14	5.02	5.31	5.68	3.72	3.65
Max	8.61	8.66	9.00	9.23	8.91	8.40	8.14	8.68	9.62	9.32
n	28.00	46.00	11.00	66.00	127.00	27.00	46.00	11.00	66.00	127.00
$LSD_{5\%}$	1.58	1.58	1.58	1.58	1.58	2.07	2.07	2.07	2.07	2.07
Variance components										
σ_G^2	1.76***	2.13***	4.42***	2.61***	2.62***	1.76***	1.66***	2.49***	2.43***	2.38***
σ_e^2	0.67	0.67	0.67	0.67	0.67	0.90	0.90	0.90	0.90	0.90
H^2	0.61	0.84	0.69	0.77	0.88	0.65	0.75	0.64	0.78	0.87
Testcrosses										
Min	1.28	1.61	1.61	1.61	1.61	1.53	1.60	1.60	1.60	1.60
Median	4.37	4.41	4.40	4.45	4.41	5.01	5.12	5.03	5.20	5.09
Mean	4.26	4.36	4.32	4.39	4.31	4.89	5.03	4.96	5.08	4.96
Max	7.50	7.50	7.50	7.50	6.34	7.86	7.86	7.86	7.86	6.95
n	34.00	57.00	113.00	105.00	163.00	34.00	57.00	113.00	106.00	163.00
$LSD_{5\%}$	1.17	1.17	1.17	1.17	1.17	1.40	1.40	1.40	1.40	1.40
Variance components										
σ_G^2	0.71***	0.29*	0.62***	0.41***	0.22***	0.90***	0.20	0.58***	0.46***	0.22***
$\sigma_{G\times L}^2$	0.42***	0.49***	0.25***	0.06	0.00	0.45***	0.57***	0.30***	0.00	0.00
σ_e^2	0.56	0.56	0.56	0.64	0.64	0.78	0.78	0.78	0.87	0.87
H^2	0.87	0.67	0.89	0.81	0.61	0.89	0.52	0.88	0.82	0.63

* $p < 0.05$; ** $p < 0.01$; *** $p < 0.001$

Supplementary Table 3b Statistics summary and variance components for NCLB mean ($NCLB_m$) in Europe. The table includes the minimum (Min), median, mean, maximum (Max.) BLUEs for each family within a trial. Number of genotypes (n), least square of a difference (LSD), genetic variance (σ_G^2), genotype and year interactions ($\sigma_{G\times Y}^2$), genotype and location interactions ($\sigma_{G\times L}^2$), genotype and year and location interactions ($\sigma_{G\times Y\times L}^2$), residuals ($\sigma_e^2$) and heritability according to Cullis et al. (2006) (H^2) to each population

Europe	$NCLB_m$ (1-9)						
	Trial 1			Trial 2		Trial 3	Trial 4
	T1xA1	T1xA2	T1xA10	T2xA3	T2xA4	T2xA5	T5xA11
Per se							
Min	-	-	-	0.65	0.65	1.18	1.18
Median	-	-	-	2.18	1.93	2.17	2.46
Mean	-	-	-	2.22	2.08	2.47	2.68
Max	-	-	-	5.43	5.51	6.47	6.47
n	-	-	-	95.00	150.00	108.00	128.00
$LSD_{5\%}$	-	-	-	0.73	0.73	0.98	0.98
σ_G^2	-	-	-	0.33***	0.70***	0.40***	1.07***
$\sigma_{G\times Y}^2$	-	-	-	-	-	0.13***	0.13**
σ_e^2	-	-	-	0.25	0.23	0.75	0.59
H^2	-	-	-	0.40	0.84	0.57	0.86
Testcrosses							
Min	1.65	1.65	1.65	1.65	1.68	1.68	1.46
Median	2.93	2.91	2.92	2.91	2.89	3.15	3.13
Mean	3.01	3.01	3.02	3.02	3.01	3.18	3.17
Max	5.18	5.18	5.18	5.18	5.18	5.04	5.04
n	33.00	59.00	110.00	107.00	148.00	93.00	102.00
$LSD_{5\%}$	0.76	0.76	0.76	0.76	0.76	0.76	0.76
σ_G^2	0.17***	0.18***	0.31***	0.29***	0.12***	0.13	0.21***
$\sigma_{G\times Y}^2$	0.00	0.04***	0.00	0.03**	0.04***	0.26**	0.16***
$\sigma_{G\times L}^2$	0.06*	0.07***	0.05	0.10***	0.06***	0.09***	0.03
$\sigma_{G\times Y\times L}^2$	0.02	0.06*	0.03***	0.05***	0.02	-	-
σ_e^2	0.31	0.31	0.31	0.32	0.32	0.34	0.30
H^2	0.75	0.78	0.90	0.89	0.71	0.68	0.80

* $p < 0.05$; ** $p < 0.01$; *** $p < 0.001$

Supplementary Table 3c Statistics summary and variance components for NCLB final scores ($NCLB_f$) in Europe. The table includes the minimum (Min), median, mean, maximum (Max.) BLUEs for each family within a trial. Number of genotypes (n), least square of a difference (LSD), genetic variance (σ^2_G), genotype and year interactions ($\sigma^2_{G\times Y}$), genotype and location interactions ($\sigma^2_{G\times L}$), genotype and year and location interactions ($\sigma^2_{G\times Y\times L}$), residuals ($\sigma^2_e$) and heritability according to Cullis et al. (2006) (H^2) to each population

Europe	$NCLB_f$ (1-9)						
	Trial 1			Trial 2		Trial 3	Trial 4
	T1xA1	T1xA2	T1xA10	T2xA3	T2xA4	T2xA5	T5xA11
Per se							
Min	-	-	-	0.73	0.73	1.33	1.33
Median	-	-	-	2.68	2.72	3.15	3.38
Mean	-	-	-	2.60	2.73	3.49	3.69
Max	-	-	-	6.24	6.24	8.36	8.36
n	-	-	-	95.00	151.00	108.00	127.00
$LSD_{5\%}$	-	-	-	1.18	1.18	1.46	1.46
Variance comp.							
σ^2_G	-	-	-	0.53***	1.01***	0.74***	1.68***
$\sigma^2_{G\times Y}$	-	-	-	-	-	0.35	0.21
σ^2_e	-	-	-	0.48	0.48	1.57	1.30
H^2	-	-	-	0.53	0.86	0.63	0.83
Testcrosses							
Min	2.07	2.07	2.07	2.07	2.33	2.41	1.81
Median	4.15	4.11	4.15	4.14	4.08	4.58	4.54
Mean	4.23	4.24	4.23	4.27	4.24	4.55	4.50
Max	6.88	7.04	7.04	7.04	7.04	7.04	7.04
n	33.00	59.00	110.00	108.00	149.00	89.00	102.00
$LSD_{5\%}$	1.09	1.09	1.09	1.09	1.09	1.09	1.09
Variance comp.							
σ^2_G	0.41***	0.34***	0.66***	0.66***	0.32***	0.11	0.25*
$\sigma^2_{G\times Y}$	0.01	0.15***	0.00	0.04	0.02***	0.62*	0.23**
$\sigma^2_{G\times L}$	0.11	0.10***	0.03	0.10*	0.04***	0.11***	0.09
$\sigma^2_{G\times Y\times L}$	0.12*	0.13*	0.17***	0.24***	0.18***	-	-
σ^2_e	0.67	0.67	0.67	0.67	0.67	0.80	0.82
H^2	0.78	0.74	0.90	0.90	0.78	0.18	0.64

* $p < 0.05$; ** $p < 0.01$; *** $p < 0.001$

Supplementary Table 3d Statistics summary and variance components for female flowering date (FF) in Europe. The table includes the minimum (Min), median, mean, maximum (Max.) BLUEs for each population within a trial. Number of genotypes (n), least square of a difference (LSD), genetic variance (σ_G^2), genotype and year interactions ($\sigma_{G\times Y}^2$), genotype and location interactions ($\sigma_{G\times L}^2$), genotype and year and location interactions ($\sigma_{G\times Y\times L}^2$), residuals ($\sigma_e^2$) and heritability according to Cullis et al. (2006) (H^2) to each population

Europe	FF (days)						
	Trial 1			Trial 2		Trial 3	Trial 4
	T1xA1	T1xA2	T1xA10	T2xA3	T2xA4	T2xA5	T5xA11
Per se							
Min	-	-	-	-	-	74.88	74.88
Median	-	-	-	-	-	87.89	87.52
Mean	-	-	-	-	-	88.47	87.78
Max	-	-	-	-	-	105.49	105.49
n	-	-	-	-	-	104.00	121.00
$LSD_{5\%}$	-	-	-	-	-	4.55	4.55
Variance components							
σ_G^2	-	-	-	-	-	32.74***	29.18***
$\sigma_{G\times Y}^2$	-	-	-	-	-	0.00	0.00
σ_e^2	-	-	-	-	-	3.84	3.84
H^2	-	-	-	-	-	0.77	0.75
Testcrosses							
Min	60.52	60.52	60.52	60.52	65.93	65.93	65.93
Median	75.32	75.72	75.60	75.47	75.18	73.94	73.72
Mean	76.17	76.31	76.24	76.24	75.88	74.41	74.16
Max	89.85	89.85	89.85	89.85	87.64	88.14	88.14
n	32.00	57.00	100.00	103.00	142.00	93.00	80.00
$LSD_{5\%}$	3.07	3.07	3.07	3.07	3.07	3.07	3.07
Variance components							
σ_G^2	15.43***	9.44***	12.15***	21.91***	20.79	10.97***	10.45***
$\sigma_{G\times Y}^2$	0.81	0.00	0.00	0.34	0.38	0.00	0.00
$\sigma_{G\times L}^2$oc	1.27	1.88**	0.57	0.75*	1.35***	0.78***	0.00
$\sigma_{G\times Y\times L}^2$	0.05	0.29	1.33***	0.61***	0.00	0.25	1.20***
σ_e^2	0.89	0.89	0.89	1.28	1.28	0.91	0.48
H^2	0.81	0.68	0.77	0.90	0.92	0.73	0.73

* $p < 0.05$; ** $p < 0.01$; *** $p < 0.001$

Supplementary Table 3e Statistics summary and variance components for plant height (PH) in Europe. The table includes the minimum (Min), median, mean, mean, maximum (Max.) BLUEs for each family within a trial. Number of genotypes (n), least square of a difference (LSD), genetic variance (σ_G^2), genotype

and year interactions ($\sigma^2_{G\times Y}$), genotype and location interactions ($\sigma^2_{G\times L}$), genotype and year and location interactions ($\sigma^2_{G\times Y\times L}$), residuals ($\sigma^2_{e}$) and heritability according to Cullis et al. (2006) (H^2) to each population

Europe	PH (cm)						
	Trial 1			Trial 2		Trial 3	Trial 4
	T1xA1	T1xA2	T1xA10	T2xA3	T2xA4	T2xA5	T5xA11
Per se							
Min	-	-	-	-	-	127.84	127.84
Median	-	-	-	-	-	194.45	181.59
Mean	-	-	-	-	-	194.07	184.92
Max	-	-	-	-	-	262.61	252.57
n	-	-	-	-	-	111.00	130.00
$LSD_{5\%}$	-	-	-	-	-	25.58	25.58
Variance comonents							
σ^2_G	-	-	-	-	-	420.04***	465.21***
$\sigma^2_{G\times Y}$	-	-	-	-	-	83.27*	52.54*
σ^2_e	-	-	-	-	-	53.39	53.39
H^2	-	-	-	-	-	0.71	0.79
Testcrosses							
Min	206.63	206.63	206.63	206.63	220.61	220.02	163.69
Median	261.82	261.87	261.86	261.85	259.83	257.73	257.00
Mean	262.15	262.28	262.09	261.93	260.65	257.71	257.00
Max	306.54	304.92	304.92	301.83	299.83	299.83	299.83
n	33.00	58.00	101.00	108.00	147.00	93.00	80.00
$LSD_{5\%}$	24.94	24.94	24.94	24.94	24.94	24.94	24.94
Variance components							
σ^2_G	236.89***	114.71**	130.55***	149.01***	141.22***	77.59*	120.97**
$\sigma^2_{G\times Y}$	0.00	0.00	0.00	0.00	14.88	0.00	1.58
$\sigma^2_{G\times L}$oc	55.20	87.09*	57.34**	15.94	64.66***	70.92*	27.33
$\sigma^2_{G\times Y\times L}$	0.00	0.00	0.00	31.29	0.00	18.08	30.59
σ^2_e	133.63	133.63	133.63	138.64	138.64	149.27	106.19
H^2	0.89	0.76	0.80	0.84	0.85	0.65	0.78

* $p < 0.05$; ** $p < 0.01$; *** $p < 0.001$

Supplementary Table 4 Phenotypic Pearson correlations (r_p) for final NCLB scores ($NCLB_f$, 1-9), mean of NCLB scores ($NCLB_m$, 1-9), days to female flowering (FF, days), plant height (PH, cm), number of genotypes per population and total number of genotypes included in each each trait comparison (n_{total})

Group	Main	Input1	Input2	r_p	Signif.	Donor	n_{total}
Europe	TC	$NCLB_f$	$NCLB_m$	0.98	***	T1	202
Europe	TC	$NCLB_f$	$NCLB_m$	0.98	***	T2	255
Europe	TC	$NCLB_f$	$NCLB_m$	0.95	***	T5	102
Europe	TC	$NCLB_f$	FF	-0.52	***	T1	199
Europe	TC	$NCLB_f$	FF	-0.53	***	T2	245
Europe	TC	$NCLB_f$	FF	-0.38	***	T5	80
Europe	TC	$NCLB_f$	PH	-0.34	***	T1	201
Europe	TC	$NCLB_f$	PH	-0.13	*	T2	255
Europe	TC	$NCLB_f$	PH	-0.13		T5	80
Europe	PS	$NCLB_f$	$NCLB_m$	0.88	***	T2	245
Europe	PS	$NCLB_f$	$NCLB_m$	0.96	***	T2×A5	105
Europe	PS	$NCLB_f$	$NCLB_m$	0.97	***	T5	125
Europe	PS	$NCLB_f$	FF	-0.41	***	T2×A5	100
Europe	PS	$NCLB_f$	FF	-0.40	***	T5	118
Europe	PS	$NCLB_f$	PH	-0.03		T2×A5	107
Europe	PS	$NCLB_f$	PH	-0.02		T5	127
Europe	$NCLB_f$	TC	PS	0.37	***	T2	242
Europe	$NCLB_f$	TC	PS	0.61	***	T5	95
Europe	FF	TC	PS	0.82	***	T5	69
Europe	PH	TC	PS	0.40	***	T5	75
Brazil	TC	$NCLB_f$	$NCLB_m$	0.98	***	T1	204
Brazil	TC	$NCLB_f$	$NCLB_m$	0.90	***	T2	268
Brazil	PS	$NCLB_f$	$NCLB_m$	0.91	***	T1	85
Brazil	PS	$NCLB_f$	$NCLB_m$	0.99	***	T2	203
Brazil	$NCLB_f$	TC	PS	0.42	***	T1	83
Brazil	$NCLB_f$	TC	PS	0.46	***	T2	201
NCLBf	TC	Brazil	Europe	0.36	***	T1	198
NCLBf	TC	Brazil	Europe	0.41	***	T2	243
NCLBf	DH	Brazil	Europe	0.44	***	T2	190

p>0.05 * $p < 0.05$; ** $p < 0.01$; *** $p < 0.001$

Supplementary Table 5 QTL identified for each resistance donor, trait, evaluation type, region, type of model used for the analysis and its respective chromosome bin (e.g.: chromosome bin 2.07 refers to chromosome 2 in the bin region 2.07), genetic position in basepairs (Mbp) and in centiMorgan (putcM), range of the superior and inferior confidence interval positions (Range), LOD score, percentage of explained genotypic variance (p_G (%)) and donor of the indicated allele substitution effect (α-effect). Final NCLB scores ($NCLB_f$), mean of NCLB scores ($NCLB_m$), days to female flowering, plant height were the traits assessed in line *per se* (PS) and testcross (TC) combinations in Europe (EU) and Brazil (BR). The traits were assessed with the bi-allelic (biall) models for donors T1 and T2, and donor T5 in a biparental analysis (bi-par) as indicated on the "Model" column. QTL nomenclature indicated on the column "QTL" presents "q" and a crescent number according to the sequence of chromosomes and genetic position. QTL with the same nomenclature indicate co-localization of a QTL within confidence interval range

Donor	Type	Region	Model	QTL	Marker	Bin	Pos (Mbp)	Pos (putcM)	Range (putcM)	LODscore	P_G (%)	α-effect
$NCLB_f$												
T2	PS	EU	biall	q1	PZE-102123692	2.07	172.38	126.03	19.35	4.25	10.01	0.55
T2	PS	EU	biall	q2	PZE-103040814	3.04	36.69	70.47	39.85	4.24	11.01	-0.58
T2	PS	BR	biall	q3	SYN34468	5.04	168.93	121.19	20.13	5.37	10.87	-1.15
T2	PS	BR	biall	q4	PZE-107098889	7.03	153.88	130.80	18.72	6.27	13.29	-1.24
T2	PS	BR	biall	q5	PZE-109064251	9.04	107.36	87.35	2.43	5.1	10.95	-1.11
T2	PS	EU	biall	q6	PZE-109078583	9.04	126.98	105.41	27.99	4.81	15.84	-0.70
T2	PS	EU	biall	q7	PZE-110049100	10.04	91.97	80.56	1.20	4.07	10.09	-0.58
T5	PS	EU	bi-par	q8	PZE-101176725	1.07	221.54	231.29	10.13	6.75	28.52	-0.70
T5	PS	EU	bi-par	q9	SYN4732	2.02	9.36	35.03	3.55	10.47	21.28	-0.62
T1	TC	EU	biall	q10	PZE-101187496	1.08	232.59	250.12	3.86	3.74	3.57	0.54
T1	TC	EU	biall	q11	PZE-104016913	4.03	16.87	52.47	35.50	4.66	12.27	0.54
T1	TC	BR	biall	q12	PZE-104104798	4.08	181.09	150.47	111.14	4.95	8.45	0.52
T1	TC	BR	biall	q13	PZE-105090729	5.04	127.64	105.84	1.91	4.56	12.41	-0.65
T1	TC	EU	biall	q14	SYN3872	8.05	131.05	121.12	13.34	8.32	18.57	-0.57
T1	TC	EU	biall	q14	PUT-163a-4647150-2111	8.08	172.67	189.54	209.86	3.87	6.57	0.41
T2	TC	EU	biall	q4	PZE-107100579	7.03	155.11	131.77	16.96	9.57	16.83	-0.59
T2	TC	EU	biall	q15	PZE-108038275	8.03	61.99	71.31	22.85	4.24	7.73	-0.34
T2	TC	EU	biall	q5	PZE-109058129	9.03	100.37	84.42	8.19	4.79	7.10	-0.37
T2	TC	BR	biall	q5	PZE-109065486	9.04	108.35	88.85	10.81	5.06	5.43	-0.30
T2	TC	EU	biall	q7	PZE-110049734	10.04	93.65	81.68	11.99	4.67	24.02	-0.74
T5	TC	EU	bi-par	q8	SYN38722	1.07	214.94	225.44	18.24	6.56	15.75	-0.29

Supplementary Table 5 (continued)

Donor	Type	Region	Model	QTL	Marker	Bin	Pos (Mbp)	Pos (putcM)	Range (putcM)	LODscore	PG (%)	α-effect
T5	TC	EU	bi-par	q16	SYN26598	2.04	41.14	94.3	8.19	7.57	17.7	-0.32
T5	TC	EU	bi-par	q17	SYN32683	6.01	93.16	49.4	10.81	8.85	30.98	-0.42
NCLB$_m$												
T5	PS	EU	bi-par	q8	SYN29307	1.03	190.59	182.04	15.49	7.98	28.21	-0.58
T5	PS	EU	bi-par	q9	SYN4732	2.02	9.36	35.03	3.55	10.09	18.1	-0.46
T2	PS	EU	biall	q1	PZE-102123692	2.06	172.38	126.03	19.93	5.29	13.27	0.47
T1	PS	BR	biall	q2	SYN10993	3.04	113.44	90.05	6.29	4.64	46.96	-1.98
T2	PS	EU	biall	q2	PZE-103038295	3.04	32.99	68.88	46.13	4.11	11.05	-0.43
T1	PS	BR	biall	q18	PZE-104153704	4.1	238.82	230.08	26.24	3.75	21.99	1.32
T2	PS	BR	biall	q3	SYN34468	5.04	168.93	121.19	20.13	5.49	10.63	-1.15
T2	TC	BR	biall	q19	PZE-102030524	2.02	14.28	51.40	8.41	4.73	11.38	0.39
T5	TC	EU	bi-par	q16	PZE-102062478	2.04	41.31	94.66	17.68	6.56	19.98	-0.32
T1	TC	EU	biall	q11	PZE-104017733	4.03	17.52	58.39	44.2	3.98	13.81	0.40
T1	TC	BR	biall	q12	PZE-104127796	4.09	207.13	186.4	21.08	6.00	8.31	0.53
T1	TC	BR	biall	q13	PZE-105090729	5.04	127.64	105.84	4.70	4.60	12.65	-0.66
T2	TC	EU	biall	q20	SYN34122	5.05	193.17	159.49	19.30	4.50	7.06	-0.24
T5	TC	EU	bi-par	q21	SYN32683	6.02	93.16	49.40	7.08	6.52	17.28	-0.27
T2	TC	EU	biall	q4	PZE-107100579	7.03	155.11	131.77	14.67	8.81	16.13	-0.37
T2	TC	BR	biall	q4	SYN14658	7.03	139.64	112.74	38.05	4.09	21.55	-0.56
Female flowering												
T5	PS	EU	bi-par	q16	PZE-102073497	2.04	54.42	107.53	12.54	8.30	17.41	2.24
T5	PS	EU	bi-par	q7	PZE-110057433	10.04	110.22	86.53	12.66	20.30	51.39	2.24
T1	TC	EU	biall	q22	PZE-102171211	2.08	215.28	181.41	1.81	5.75	2.79	1.15
T2	TC	EU	biall	q23	PZE-107137797	7.06	175.41	205.18	36.81	12.34	3.98	1.92
T2	TC	EU	biall	q14	PZE-108069804	8.04	122.28	111.21	20.20	8.27	3.49	1.77
T1	TC	EU	biall	q14	PZE-108098580	8.06	154.54	132.94	3.64	4.26	2.83	1.18
T2	TC	EU	biall	q24	PZE-109000639	9.00	1.26	2.28	3.73	4.26	1.21	1.34
T1	TC	EU	biall	q5	PZE-109030065	9.03	34.72	73.54	49.21	7.94	7.61	1.70
T2	TC	EU	biall	q6	PZE-109085291	9.05	134.20	123.8	23.27	4.04	1.19	1.13
T2	TC	EU	biall	q7	PZE-110013181	10.02	11.81	52.73	36.00	4.74	0.65	0.92

Supplementary Table 5 (continued)

Donor	Type	Region	Model	QTL	Marker	Bin	Pos (Mbp)	Pos (putcM)	Range (putcM)	LODscore	PG (%)	α-effect
T1	TC	EU	biall	q7	PZE-110050499	10.04	95.22	82.33	5.93	22.6	96.26	6.54
T2	TC	EU	biall	q7	PZE-110050010	10.04	94.21	81.91	0.58	18.17	55.03	8.03
T5	TC	EU	bi-par	q7	PZE-110057433	10.04	110.22	86.53	12.66	24.7	84.02	3.29
Plant height												
T5	PS	EU	bi-par	q5	PZE-109029741	9.03	33.77	73.45	20.66	8.05	17.48	8.70
T2	TC	EU	biall	q25	PZE-102149586	2.07	196.98	145.31	27.23	4.46	6.44	6.67
T2	TC	EU	biall	q23	PZE-107137797	7.06	175.41	205.18	3.20	4.16	5.52	6.42
T2	TC	EU	biall	q14	SYN32800	8.05	124.33	117.51	20.20	4.38	6.94	7.19
T2	TC	EU	biall	q7	SYN6300	10.03	80.73	71.69	19.45	7.32	37.34	16.92
T1	TC	EU	biall	q7	PZE-110060686	10.04	114.89	91.48	28.34	4.42	33.70	16.10
T5	TC	EU	bi-par	q7	PZE-110048295	10.04	90.49	80.32	20.12	8.48	26.79	8.60

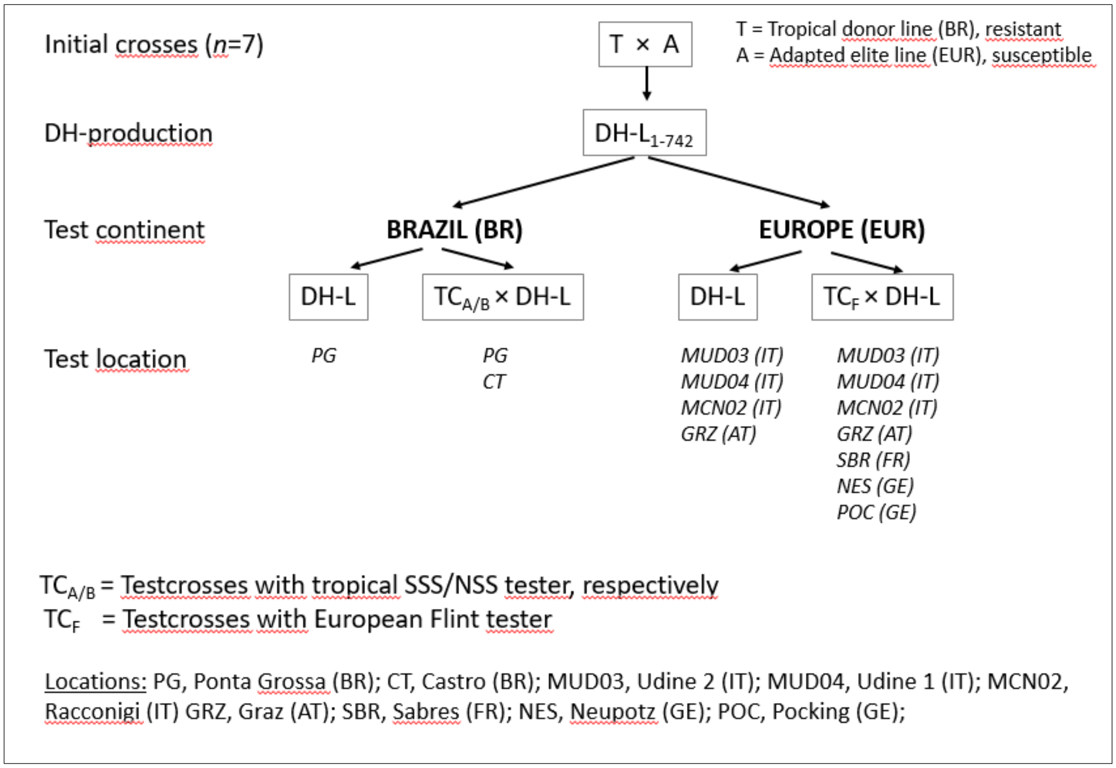

Supplementary Fig. 1 Schematic illustration of the genetic materials and testing locations

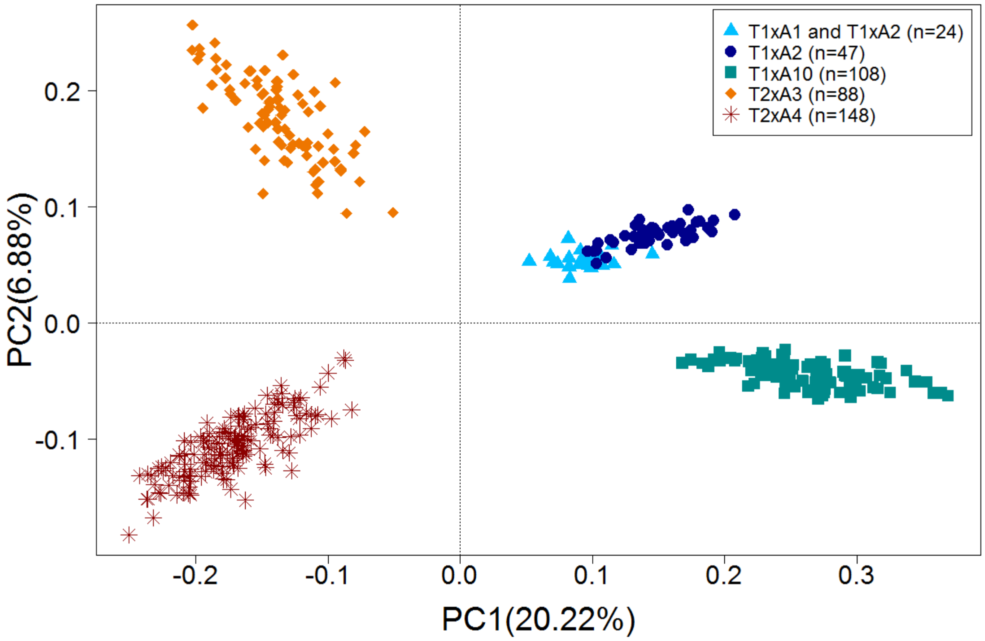

Supplementary Fig. 2 Principal component (PC) analysis based on modified Rogger's distance for populations T1×A1, T1×A2, T1×A10, T2×A3 and T2×A4, where the prefixes "T" and "A" are assigned to "tropical" and "adapted" double haploid (DH) parents, respectively. Populations T2×A5 and T5×A11 were not included in the PC due to the low number of overlapping markers with the presented population

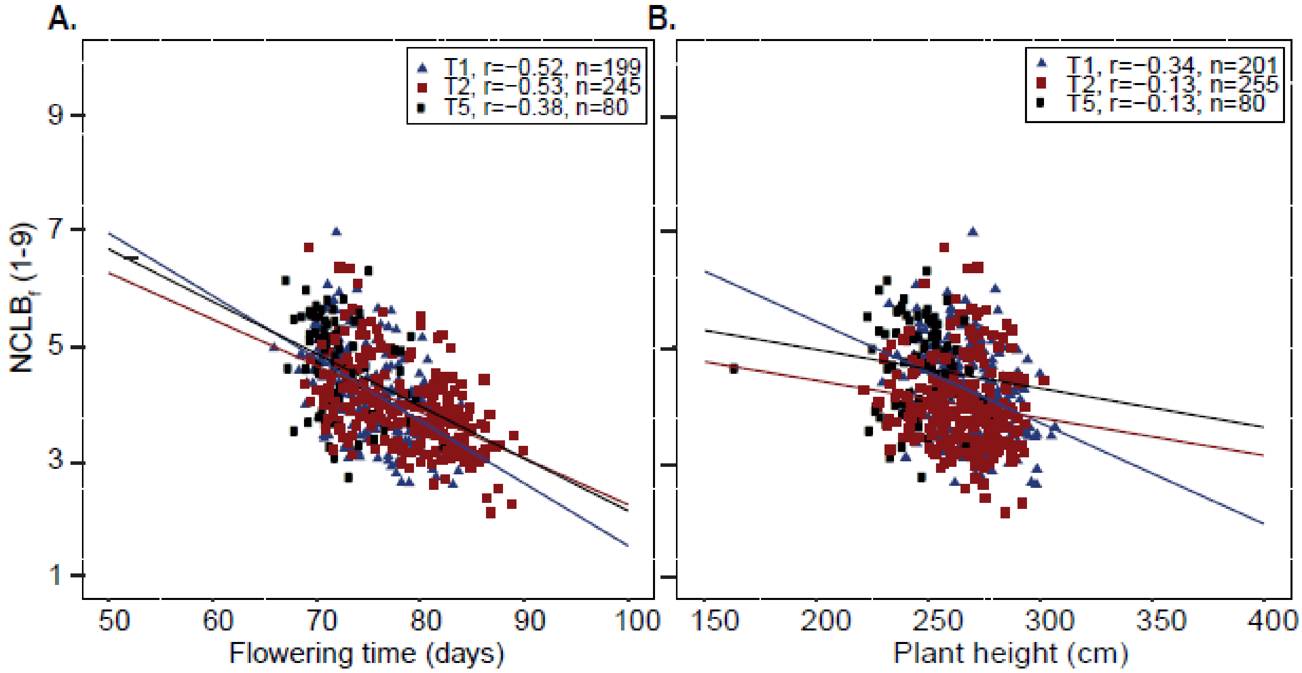

Supplementary Fig. 3 Scatter plots for final NCLB score ($NCLB_f$) and flowering time evaluated as testcrosses in Europe (a) and $NCLB_f$ plotted against plant height evaluated as testcrosses in Europe (b) as well as the phenotypic correlation (r) and number of genotypes (n)
